# A Comparative Analysis of Intratesticular Versus Intra‐Epididymal Injections of Clove Essential Oil on Male Dog Sterilization

**DOI:** 10.1002/vms3.70749

**Published:** 2025-12-30

**Authors:** Morteza Poormohammad, Mohammad Hossein Safari, Shadi Emami Moghadam, Ourang Ataie Amarloie, Mehran Farhoodi Moghadam, Pegah Valitabar, Fariborz Moayer

**Affiliations:** ^1^ Faculty of Veterinary Medicine Karaj Branch Islamic Azad University Karaj Iran; ^2^ Department of Internal Medicine Faculty of Veterinary Medicine University of Tehran Tehran Iran; ^3^ Department of Clinical Sciences Faculty of Veterinary Medicine Karaj Branch Islamic Azad University Karaj Iran; ^4^ Department of Avian Diseases Faculty of Veterinary Medicine University of Tehran Tehran Iran; ^5^ Department of Pathobiology Faculty of Veterinary Medicine Karaj Branch Islamic Azad University Karaj Iran

**Keywords:** clove, dog, injection, intra‐epididymal, intratesticular, sterilization

## Abstract

**Background:**

Chemical castration offers a highly beneficial and safe alternative to surgical sterilization for stray male dogs.

**Objective:**

This study investigates the effectiveness of intratesticular (ITI) and intra‐epididymal (IEI) injections of clove essential oil for chemical castration in male dogs as a non‐surgical sterilization method.

**Materials and Methods:**

Twenty‐four mixed‐breed male dogs were randomly assigned to four groups: two treatment groups receiving clove‐oil injections (bilateral ITI or IEI) and two control groups receiving saline. Blood samples were collected on Days 0, 3, 12, 21, 30 and 40, whereas semen quality and ultrasound data were analysed at 8‐ and 4‐day intervals, respectively, to assess testosterone levels, sperm quality and testicular and epididymal changes. Dogs were castrated on Day 40 for morphological and histological tissue evaluation.

**Results:**

Both treatment groups showed significant decreases in sperm concentration, motility and viability and increased sperm abnormalities compared to control groups (*p* < 0.05). ITI injection caused significantly greater testosterone decline (*p* < 0.005), whereas IEI treatment produced notably enlarged epididymal tail volumes (*p* < 0.0001). Histological evaluation demonstrated marked testicular necrosis, inflammation and fibrosis following ITI clove‐oil administration, whereas IEI alterations remained mild. Ultrasound assessments confirmed altered blood flow and echogenicity consistent with reproductive tissue damage after ITI injection.

**Conclusions:**

Clove‐oil injections could provide an effective, less invasive alternative to surgical castration. ITI administration was simpler and produced permanent testicular damage, whereas IEI treatment induced mainly functional changes with minimal structural disruption by Day 40. Confirming reversibility of epididymal effects will require comprehensive long‐term investigation.

## Graphical abstract

This study evaluated clove essential oil as a non‐surgical sterilant in male dogs. Both intratesticular and intra‐epididymal injections significantly reduced sperm concentration, motility and viability, with histological and sonographic evidence of reproductive tissue damage. The intratesticular route proved more effective, highlighting clove oil as a practical alternative for controlling stray dog populations.

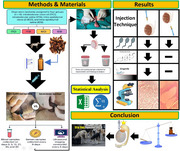

## 1 Introduction

Dogs are widely found across the globe due to their friendly demeanour and close bond with humans. However, controlling their reproduction presents significant obstacles, especially in managing stray dog populations. Effective population control requires a variety of approaches tailored to specific regions, along with thorough assessment and intervention strategies. The high number of stray dogs is a global issue, largely resulting from inadequate sterilization rates (FAO 2014). Research indicates that even with 100% annual sterilization of known stray dogs, achieving an overall rate above 86% after 20 years remains unrealistic (Dias et al. 2015). This overabundance results in uncontrolled breeding, highlighting the need for effective sterilization techniques (Peña‐Corona et al. 2022).

Sterilization, which renders animals infertile, is a longstanding method for regulating dog breeding (Moldave and Rhodes 2013). Although surgical sterilization is prevalent, it carries risks, including bleeding, wound adhesions, infection, scrotal swelling and behavioural changes such as increased aggression (Adin 2011; Neilson et al. 1997). In contrast, non‐surgical methods offer advantages such as ease of implementation, lower cost, high effectiveness and minimal health risks. They also allow policymakers and animal shelters to manage resources more efficiently. An ideal non‐surgical sterilization technique should suppress fertility and reduce unwanted sexual behaviours, including aggression. Several chemical methods, including hormonal treatments and immune‐contraceptives, have been investigated (M. Kutzler and Wood 2006). Safe phytosterols and synthetic chemicals that suppress spermatogenesis and sexual drive provide relatively simple, low‐cost and less invasive alternatives to surgery, while avoiding complications such as bleeding, hernias and infections. Over the past 50 years, a variety of inorganic chemical compounds—including cadmium carbide, iron chloride, sodium chloride, danazol, Bacillus Calmette–Guerin (BCG), glycerol and formalin—have been evaluated for sterilizing laboratory and domestic animals. These methods have shown potential but may cause temporary side effects such as mild scrotal pain, swelling, redness, fatigue and diarrhoea, typically resolving within a week (Canpolat et al. 2016; Das et al. 1982; Falah Mahmood Hameed et al. 2024; Ijaz et al. 2000; Immegart and Threlfall 2000; Mohamed et al. 2024).

Chemical sterilization can be administered via intraperitoneal, intratesticular (ITI) and intra‐epididymal (IEI) routes. Among these, ITI and IEI injections are particularly effective because they directly target spermatogenesis and sperm transit, while leaving other aspects of fertility unaffected (Leoci et al. 2019; Mobarak et al. 2022). The epididymis—comprising the head (housing immature sperm) and the tail (housing mature sperm)—is crucial for sperm maturation and storage, making it a key target for injection‐based sterilization (Igdoura and Wiebe 1994; Jones 1999). A recent comprehensive review highlighted the historical evolution of castration practices, evaluated current methods and emphasized the need for low‐invasiveness alternatives. These findings underscore the importance of exploring chemical sterilants and advancing non‐surgical contraceptive strategies in dogs and cats as lower invasiveness approaches to population control that are associated with reduced surgical burden (Hess et al. 2024; Peña‐Corona et al. 2025). Furthermore, contrast‐enhanced ultrasound has recently been used to characterize vascular alterations following ITI calcium chloride injection in dogs, providing detailed insight into testicular perfusion changes associated with chemical castration (Cicirelli et al. 2022).

Clove essential oil (*Eugenia caryophyllata*), obtained from *Syzygium aromaticum*, is widely used in human medicine as an anaesthetic, sedative and flavouring agent. It is cultivated in countries such as Indonesia, Madagascar, India and Sri Lanka (Wynn and Fougère 2007). In veterinary medicine, clove oil exhibits strong antibacterial and antifungal properties (Calderone et al. 1994). Eugenol, the primary component (70%–90%), is commonly applied in dentistry as an analgesic for toothache relief (Chung and Oh 2013). At lower concentrations, eugenol provides antioxidant and anti‐inflammatory benefits, whereas at higher concentrations, it can act as an oxidant, leading to tissue necrosis through free radical generation (Kumar Jaganathan and Supriyanto 2012; Moleyar and Narasimham 1992). Recent studies have also explored chemical and plant‐based sterilants as practical alternatives to surgical castration, showing potential for population control in stray dog populations (Peña‐Corona, León‐Ortiz, et al. 2022).

This study aims to evaluate the sterilizing efficacy of ITI versus IEI clove‐oil administration in male dogs, focusing on sperm viability, testicular necrosis, morphological changes (testis/epididymis histology) and haemodynamic alterations (testicular blood flow). Furthermore, it seeks to determine the optimal administration route for non‐surgical sterilization in stray dog populations, based on safety and efficacy outcomes.

## 2 Methods and Materials

### 2.1 Animals

Twenty‐four mixed‐breed male dogs (24–36 months, mean 28.83 ± 0.68 months; 16–24 kg, mean 19.46 ± 0.42 kg; body condition score 4.67 ± 0.16) were obtained from shelters in Alborz Province and transported to Rakhsh Large Animal Hospital (Latitude: 35°59′29.6″N; Longitude: 50°37′24.6″E Decimal: 35.991559, 50.623501). Dogs were physically healthy with normal testicular and epididymal structures confirmed by ultrasound and exhibited normal sexual behaviour. Semen parameters were within reference ranges (concentration ≥300 × 10^6^/mL, motility ≥70%, viability ≥80% and total abnormalities <20%) and serum testosterone ≥1 ng/mL (Purswell et al. 2010). The animals were placed in conventional kennels common to the type used for dog boarding services. The facility's temperature, humidity and overall ventilation were satisfactory, and washing and disinfection amenities were effectively provided (Cavill 2008). Dogs were housed in ventilated kennels, vaccinated against rabies (BIOCAN R, Bioveta a.s., Czech Republic) and treated with antiparasitic tablets (Droncomplex, Topkim, Turkey) at 15 mg/kg. Water was provided ad libitum, and each dog received 245–350 g/day of standardized dry food (Nutriepet, 21% protein, Iran) with multivitamin supplementation (Beaphar, Top Ten, the Netherlands).

### 2.2 Experimental Protocol

Dogs were randomly assigned to four groups (*n* = 6): ITI clove oil (ITCI), ITI saline (ITSI), IEI clove oil (IECI) and IEI saline (IESI). Depending on the injection method, clove oil or saline was administered bilaterally. Each treatment group (ITCI and IECI) was compared with its corresponding control group (ITSI and IESI). Blood samples were collected on Days 0, 3, 12, 21, 30 and 40 to assess serum testosterone levels (see Section 2.7). Semen samples were collected from each group every 8 days for analysis (see Section 2.6). Ultrasound imaging was conducted every 4 days to evaluate the circulatory system of the testes and the structural appearance of the testicles and epididymal tail, including changes in shape, size and tissue damage (see Section 2.8). Dogs were castrated on D40, and their testes and epididymal tails were collected for morphometric and histological analysis (see Sections 2.9 and 2.10).

### 2.3 Preparation and Extraction of the Clove Plant

Fresh clove buds (*S. aromaticum*) of 1 kg were sourced from a designated research planting site in Kashan County, coordinated with the Agricultural Jihad Department of Isfahan Province (Latitude: 32°36′17.0″N; Longitude: 51°39′47.6″E; Decimal: 32.604719, 51.663232). After species confirmation, the buds were transported to food chemistry laboratory of the Faculty of Veterinary Medicine, Islamic Azad University, Karaj Branch (Latitude: 35°51′29.1″N; Longitude: 50°59′18.7″E; Decimal: 35.858087, 50.988540). The clove buds were dried in the dark without the application of heat and subsequently ground into clove powder using a laboratory mill (KTG lab group, Iran). The Soxhlet method was employed for essential oil extraction. For this purpose, 25 g of clove powder was wrapped in cartridge paper (Whatman, China) and placed in the Soxhlet apparatus (KTG lab group, Iran). A volume of 250 mL of 95.00% ethanol was measured in a 250 mL round‐bottom flask (KTG lab group, Iran), which was then placed on a heating mantle (KTG lab group, Iran). When water circulation through the condenser was stable, the extraction chamber was built, and the process began when ethanol was boiling. The Soxhlet extraction process was then performed for 5 h. The extracted samples were left to cool for 30 min before being taken off the heating mantle. The solvent was then analysed in a rotary evaporator (model 300 STRIKE, WIGGENS Italy) at 40°C (centigrade) and under a vacuum for 30 min after the water bath was provided. The evaporated solvent was subsequently collected in a 500 mL round‐bottom flask, whereas the remaining sample was contained in a 250 mL round‐bottom flask. The resulting clove essential oil was estimated to contain approximately 100% eugenol, the primary bioactive component. Lastly, the processed sample was aliquoted into 10 mL amber glass bottles containing data labels and then kept in a refrigerator at 4°C (Murugananthan et al. 2022; Shafira et al. 2020).

### 2.4 Injection

**FIGURE 1:**
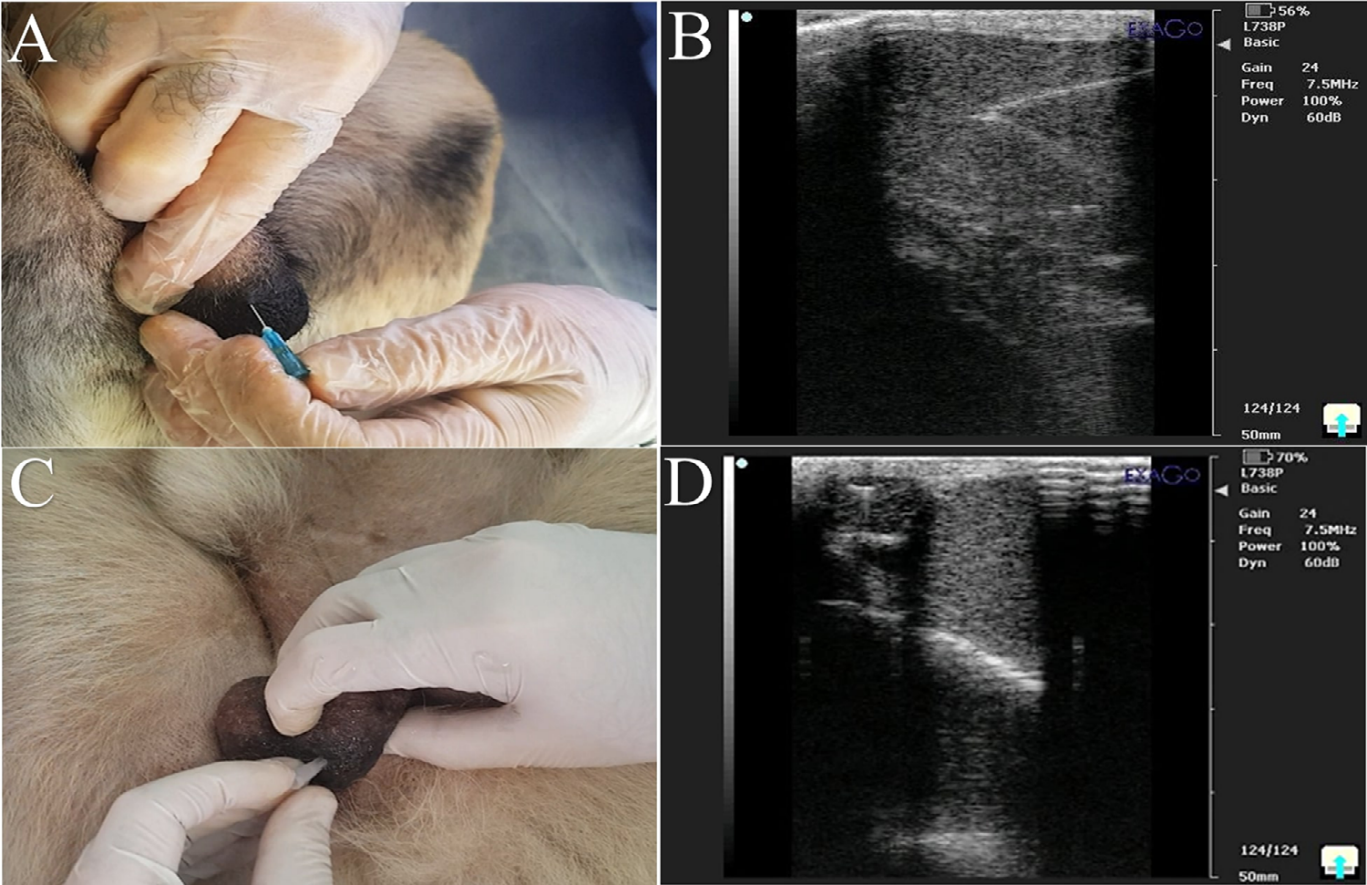
(A) Site of intratesticular injection. (B) Ultrasound view of needle in intratesticular injection. (C) Site of intra‐epididymal injection. (D) Ultrasound view of needle in intra‐epididymal injection.

The scrotal area was clipped and disinfected with 70% isopropyl alcohol (Komakol, Iran) for 30 s prior to injection. Dogs were sedated with ketamine (5 mg/kg IV, 10%, Bremer Pharma GmbH, Germany) and diazepam (0.25 mg/kg IV, Zepadic, Caspian Tamin, Iran), and anaesthesia was maintained with isoflurane (1%–2%, IsoFlo, Zoetis, USA) under continuous monitoring. Animals were positioned in lateral recumbency, and transmission gel (Poly Gel, Iran) was applied to the scrotal region. For ITI injections (ITCI and ITSI), a 24‐gauge, 2.5 cm needle (Teb Tolid, Iran) was inserted along the longitudinal axis of the testis near the epididymal head (Figure 1A,B) (Seid and Terefe 2019). IEI injections (IECI and IESI) were performed under real‐time ultrasound guidance (IVM imaging, ECM, France) using a 26‐gauge, 5–8‐in. needle, with the cauda epididymis visualized by a 7.5 MHz (megahertz) linear probe (KAIXIN Co., China; depth 2–4 cm) and needle tip placement confirmed by echogenic feedback (Figure 1C,D). Injection volumes of clove oil (ITCI) (Abshenas et al. 2013; Wynn and Fougère 2007) or normal saline (ITSI) (Pharmaceutical Solution Industry, Iran) were determined on the basis of testicular width (0.6–1.4 mL/testis; Table 1), whereas IEI volumes were set at 25% of the corresponding ITI volume (0.15–0.35 mL), reflecting the relative size of the epididymis (Leoci et al. 2019).

**TABLE 1:** Volume of intratesticular injection according to testicular width.

Testis width (mm)	Volume per testis (mL)
13–15	0.6
16–18	0.8
19–21	1
22–24	1.2
25–27	1.4

### 2.5 Clinical Observation and Behaviour Monitoring

Dogs were observed every 4 h during the first 24 h for pain (e.g., vocalization, reluctance to move), swelling or injection‐site infection. Subsequent evaluations occurred every 4 days until D12 and every 8 days until Day 40 (D40). Clinical examinations were performed 72 h post‐injection. At D40, body weight, food intake, testicular consistency, scrotal and inguinal skin condition, rectal temperature, respiratory rate, heart rate and general behaviour were assessed. Testes were palpated to determine size and hardness, with normal testes described as smooth, firm, oval‐shaped and slightly tender; the epididymis lies just behind and slightly lateral to the testicle (Walker et al. 1990). Sexual behaviours were recorded daily until D12 and then every 4 days until D40, using manual manipulation combined with sterile tampon gauze soaked in oestrous vaginal secretions from a female dog (M. A. Kutzler 2005).

### 2.6 Semen Collection and Evaluation

**FIGURE 2:**
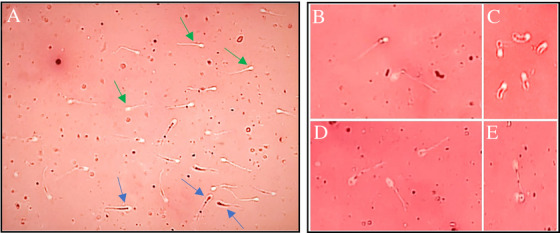
(A) The blue arrows indicate the dead sperm in which the stain has penetrated the cytoplasm, and the green arrows show the live sperm, which is not stained (Eosin–Nigrosine staining, 100×); (B) proximal cytoplasmic droplets and (C) tail slump defects are the examples of primary sperm abnormalities and (D) giant or broad heads and (E) looped tails are the examples of secondary sperm abnormalities (Eosin–Nigrosine staining, 400×).

Semen samples were collected by manual manipulation, and the sperm‐rich fraction was obtained in pre‐warmed Falcon tubes (Shimi Pajouh, Iran) at 37°C (Purswell et al. 2010). The samples were subsequently transported to the laboratory within 5 min in a 37°C water bath for evaluation of sperm parameters (Tesi et al. 2018). The evaluated parameters included semen volume, colour, concentration, total sperm count, motility, viability, sperm abnormalities (primary, secondary and total abnormalities), total sperm motility and progressive motility. Semen volume was measured using calibrated Falcon tubes (range: 1–30 mL). Semen rich in sperm typically appears milky or cloudy, whereas sperm‐free semen appears colourless and clear; this differentiation was made through direct observation. Sperm concentration (×10^6^/mL) was determined using a haemocytometer (Neubauer counting chamber) after a 1:100 dilution in normal saline, with counts performed in duplicate for each sample under a light microscope (Olympus, Tokyo, Japan) at 400× magnification. Total sperm count (×10^6^) was derived by multiplying concentration values with the measured volume. Sperm viability and morphological abnormalities were evaluated using the Eosin–Nigrosine staining method. Briefly, semen was mixed with Eosin‐Y 5% and Nigrosine 10% (Merck, Germany) on a pre‐warmed surface, smeared on glass slides (Aria ShimiAzma, Iran) and examined under a light microscope at 400× magnification. Live sperm appeared unstained, whereas dead sperm exhibited staining (Figure 2A). The viability rate was calculated using the following formula (Johnston et al. 2001):

$$\mathrm{Viability}\;\mathrm{rate}\;\left(\%\right)=\left(\mathrm{Total}\;\mathrm{unstained}\;\mathrm{sperm}/\mathrm{Total}\;\mathrm{sperm}\right)\times 100$$

Sperm abnormalities were assessed by counting 100–200 spermatozoa on a stained slide under 1000× magnification (oil immersion). Abnormalities were classified as primary (e.g., head defects) or secondary (e.g., tail coiling) per established criteria (Figure 2B–E) (Oettlé 1990). Total and progressive sperm motilities were initially assessed on pre‐warmed glass slides (37°C) under 400× magnification (Johnston et al. 2001). For detailed analysis, semen was diluted (1:5) in a TRIS extender containing 20% egg yolk (Hermansson et al. 2021), stored at 4°C and transported to a veterinary laboratory

(institution name and location withheld for anonymity) within 12 h (Hori et al. 2014). At the end, these samples were subsequently evaluated using a computer‐assisted semen analysis system (CASA; Hooshmand Fanavar Tehran Co., Iran), with the sperm chamber maintained at 37°C (Rijsselaere et al. 2012).

### 2.7 Assay for Serum Testosterone

During the specified days, at around 9:00 AM (Section 2.2), the dogs were given 1000 IU of HCG (Human Chorionic Gonadotropin, Pooyesh Darou Company, Tehran, Iran) per subcutaneous. This procedure was done to enhance testosterone production in gonads (Santana et al. 2012). Blood samples of the saphenous vein were obtained 60 min after HCG administration. Blood samples were collected both pre‐ and post‐HCG injection on D0 to map the testes’ response to stimulation and the functional efficiency of the Leydig cells. This approach enhanced the difference between physiological changes and diseases in light of other factors like stress, time of the day or general well‐being (Amador and Bartke 1987; Sharpe and Bartlett 1985). The blood samples were immediately moved to an ice container (4°C) to a veterinary laboratory. Upon arrival at the laboratory, the samples were centrifuged at 1500 × *g* (gravity) for 15 min soon after collection. The plasma obtained was centrifuged at 2000 rpm, portioned and stocked at −80°C, where a hormonal assay was to be carried out. Serum testosterone concentrations were determined using the ELISA technique (Monobind Testosterone Kit, USA) (Taya et al. 1985).

### 2.8 Ultrasound Study

**FIGURE 3:**
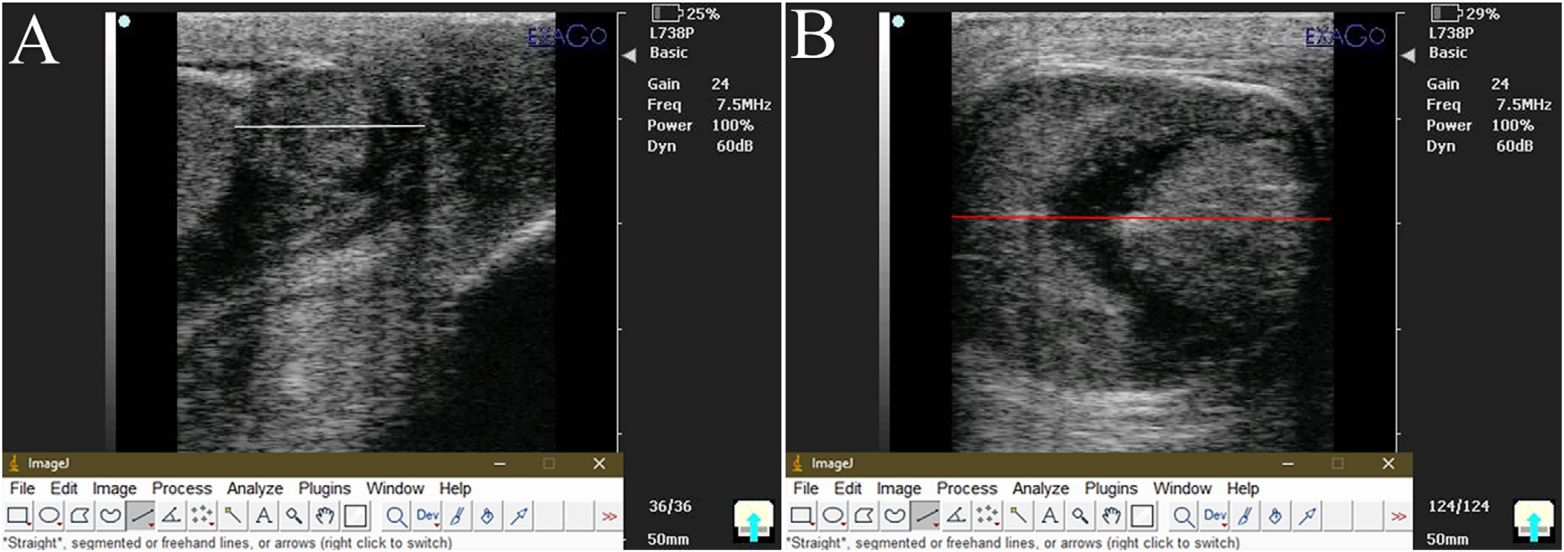
(A) B‐mode ultrasound image of epididymal tail in sagittal plane. The white line indicates epididymal tail high measurement in imageJ software; (B) B‐mode ultrasound image of testis in longitudinal plane. Red line shows testicular length measurement in imageJ software.

Ultrasound examinations of the testes and epididymal tails were performed using a real‐time B‐mode scanner (IVM imaging, ECM, France) with a 7.5 MHz linear probe (KAIXIN Co., China). Standardized settings were applied throughout the study (up to D40), with dogs positioned in lateral recumbency without sedation. After clipping scrotal hair and applying acoustic gel (Poly Gel, Iran), images were obtained in sagittal, transverse and dorsal planes and analysed using Fiji software, an improved version of ImageJ software (GPL v3 license, https://fiji.sc/) (Figure 3) (Goldstein et al. 2018; Rasband 2010; Schindelin et al. 2012). Total testicular volume (TTV) and total epididymal tail volume (TEV) were calculated using the following formulas:

$$\mathrm{TTV}\;\left(\mathrm{cubic}\;\mathrm{centimeter}-cm^{3}\right)=l\times w\times h\times 0.71$$

$$\mathrm{TEV}\;\left(\mathrm{cubic}\;\mathrm{centimeter}-cm^{3}\right)=l\times w\times h\times 0.523$$

where 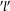 represents length (cm), 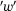 width (cm) and 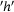 height (cm) (Gouletsou et al. 2008; Hsieh et al. 2009).

**FIGURE 4:**
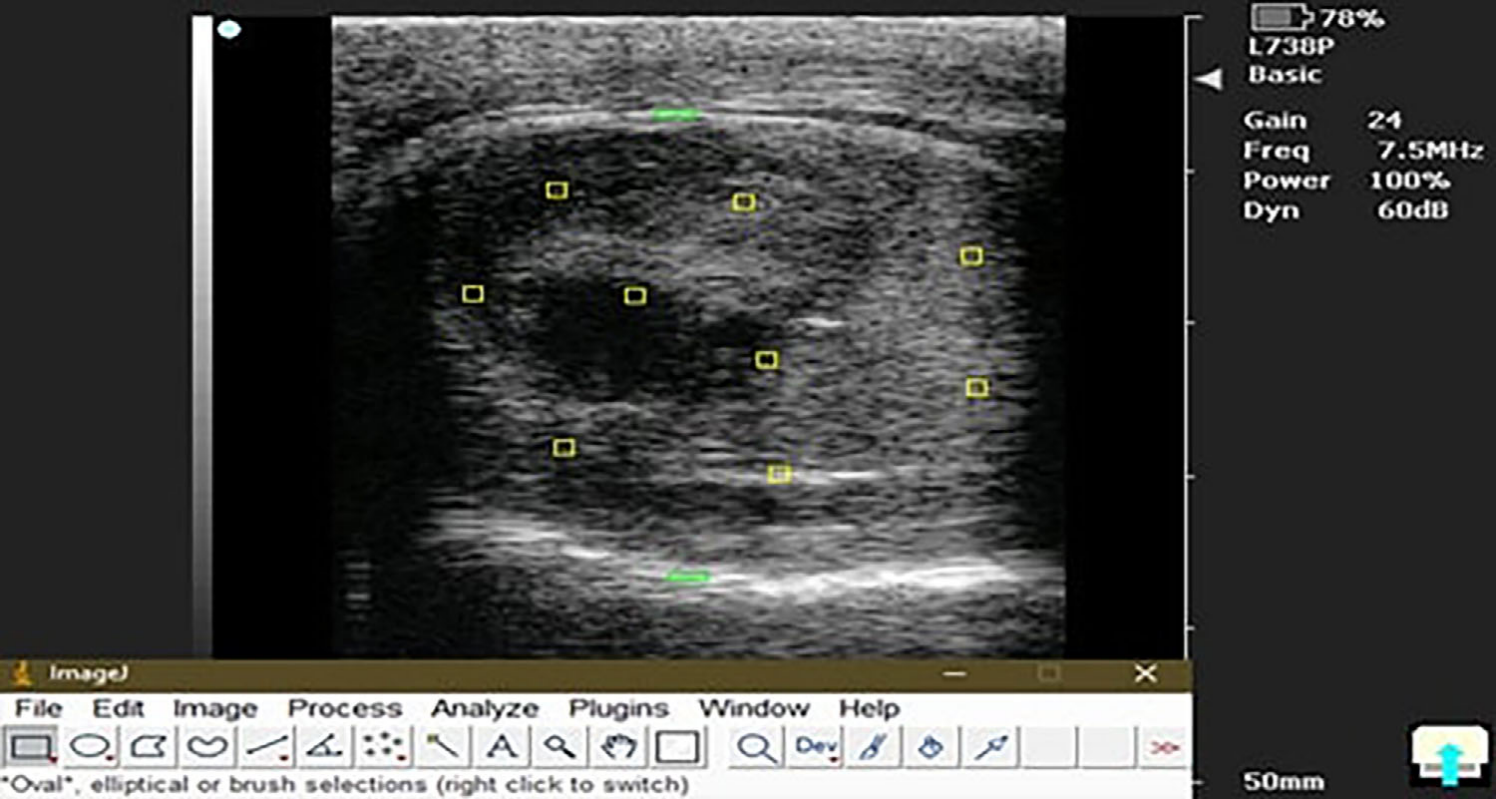
Measurement of pixel intensity in imageJ software; the two green rectangles are the near and far field echogenic reference points, and nine yellow squares represent different echogenic regions over the testicular parenchyma.

Frozen digital images of both testes in the sagittal cross‐section were analysed automatically using Fiji software to measure echogenicity. The software determined each region's mean pixel intensity (PXI), with higher percentages indicating structures of greater echogenicity. Two reference points on the testicular capsule (the most recognizable echogenic structures) were selected to measure echogenicity, one near and one far. The software randomly assigned nine sampling areas (each 2 mm^2^ in diameter) on the testicular parenchyma, avoiding the central mediastinum. The mean PXI was calculated for each region, and the reference point with the highest mean PXI (highest echogenicity) was used to compute the echogenicity. The percentage of maximum PXI was calculated using the formula (Figure 4) (Souza et al. 2015):

$$\begin{array}{ccc} \mathrm{Echogenicity}\;\left(\%\right) & = & \mathrm{Mean}\;\mathrm{PXI}\;\mathrm{of}\;\mathrm{testicular}\;\mathrm{parenchyma}/\\ & & \mathrm{Mean}\;\mathrm{PXI}\;\mathrm{of}\;\mathrm{capsule}\times 100 \end{array}$$

**FIGURE 5:**
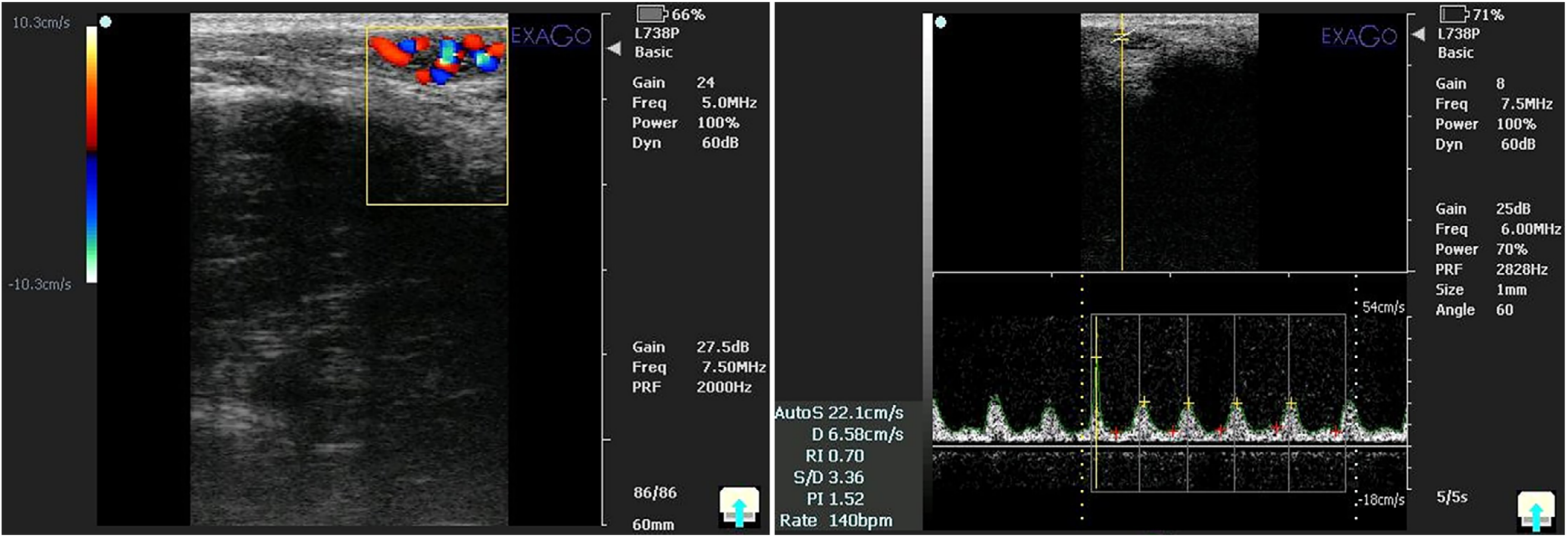
An assessment of the blood flow within the supra‐testicular artery was performed by colour‐pulsed Doppler ultrasonography, and the blood flow appeared as a spectral pattern with a wave‐like display. The blood flow parameters were calculated automatically and were displayed on screen.

Colour Doppler (CD) and pulsed‐wave (PW) spectral Doppler ultrasonography were employed to assess testicular arterial blood flow (Günzel‐Apel et al. 2001). Standardized imaging settings (depth: 1–5 cm, wall filter: 0.001 m/s, pulse repetition frequency: 2.91 kHz, insonation angle: <60°) were used to optimize measurement accuracy (Souza et al. 2015). The posterior pampiniform plexus (medial supra‐testicular region) was examined to localize the testicular artery, identified by characteristic colour flow signals, which intensified in the spermatic cord loop due to higher vascularization. Haemodynamic parameters, including peak systolic velocity (PSV), end‐diastolic velocity (EDV), resistive index (RI) and pulsatility index (PI), were calculated using the ultrasound software. Final values were derived as the mean of three consecutive waveform measurements (Figure 5) (Günzel‐Apel et al. 2001).

### 2.9 Evaluation of Morphometric Indices

**FIGURE 6:**
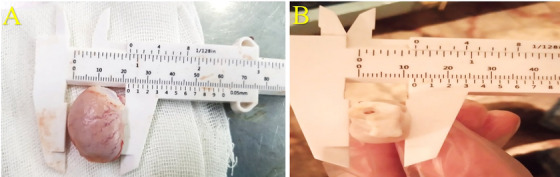
(A) Measurement of testicular height by caliper for TTV calculation; (B) measurement of epididymal tail length by caliper for TEV calculation.

On the last day as part of the study (D40), after measurement of the dogs’ weight, both testicles were surgically exposed through an open castration under general anaesthesia. The length, width and height of each testicle and epididymal tail were then measured using a caliper (Mitutoyo, Japan) (Figure 6), and the weight of each testicle (excluding the epididymis) was determined using a digital scale (Sartorius, Germany). These measurements were performed to calculate the testicular and epididymal tail volumes, employing the formula described in Section 2.8 (Junaidi and Martin 2013; Souza et al. 2015). Additionally, the Gonadosomatic Index (GSI) was calculated using the following formula (Bridges et al. 2004):

$$\begin{array}{ccc} \mathrm{GSI}\;\left(\%\right) & = & \left(\mathrm{Left}\;\mathrm{testicular}\;\mathrm{weight}+\mathrm{Right}\;\mathrm{testicular}\;\mathrm{weight}/\right.\\ & & \;\left.\mathrm{Total}\;\mathrm{body}\;\mathrm{weight}\right)\times 100 \end{array}$$

### 2.10 Prepare Slides and Evaluate Tissue Changes

The testis was incised longitudinally and, along with the epididymal tail, was immediately placed in plastic container containing 10 % buffered formalin (Arya Teb, Iran) for histological purposes. After collection, the samples were transported to the pathology laboratory of the Veterinary Medicine Faculty. These samples were embedded in paraffin moulds using a tissue preparation machine and sectioned using a microtome (KTG lab group, Iran). The sections were deparaffinized, rehydrated, stained with hematoxylin and eosin (Merck, Germany), then mounted and examined under the light microscope. A calibrated ocular lens was used to measure the diameter of seminiferous tubules, the distance between the tubes and the thickness of the germinal epithelium, and measurements were converted to the micron scale. Only tubules with circular or approximately circular shapes—those with vertical and horizontal diameter differences of less than 20%—were selected for the mean diameter calculation. The numbers of Sertoli cells, Leydig cells, spermatogonia, primary spermatocytes and spermatids were quantified in 100 randomly selected seminiferous tubules, standardized to areas of approximately 0.04 mm^2^. Counts were performed in two replicates per slide, and the arithmetic mean for each parameter was calculated and reported for each sample (Hamedi et al. 2018). FIJI software was employed to analyse stored images from each slide (Rasband 2010; Schindelin et al. 2012). Johnsen's scoring system was utilized for a more detailed evaluation to assess and score the samples (Johnsen 1970).

### 2.11 Collection of Results and Statistical Analysis

All data were recorded in an Excel file, and statistical analyses, as well as comparisons of group averages, were performed using IBM SPSS Statistics 27 software. The compatibility of the data was evaluated using the Shapiro–Wilk test to check the data's normality. A repeated measures analysis of variance (two‐way ANOVA) was conducted to assess differences in sperm, testosterone, ultrasound and Doppler parameters over time within each group, and finally, the Bonferroni post hoc test was conducted. To evaluate differences in morphometric and histopathological measurements between untreated control groups (ITSI, IESI) and treated experimental groups (ITCI, IECI) on D40, we performed parametric paired *t*‐tests for normally distributed data and non‐parametric Mann–Whitney *U* tests for non‐normally distributed datasets. Catalogue data were analysed using a significance level of *p* < 0.05. On the other hand, the mean and standard error were computed using equations on the Excel sheet. All the data analysis was done in Excel, and the results were presented in charts or graphs as appropriate.

## 3 Results

### 3.1 Clinical Outcomes and Local Reactions

Body weight (19.08 ± 0.41 kg) and vital signs—including rectal temperature, respiration rate and heart rate—remained stable throughout the study, with no significant differences observed between groups. No respiratory distress or abdominal contractions were noted after injections. At 1‐week post‐injection, no skin inflammation or other overt side effects were observed at the injection sites. Mild localized inflammation was present in the ITCI and ITSI groups, within the testicular tissue and in the caudal epididymis of the IECI group for 72–96 h post‐injection. Additionally, increased tissue hardness was noted at 24 h—persisting up to 5–6 days—in the ITCI (testicular tissue) and IECI (epididymal tissue) groups. Despite these findings, the dogs did not display discomfort upon palpation. Sexual behaviour in the ITCI group deteriorated over the trial, with no sexual interaction observed on Days 36 and 40. In contrast, the ITSI, IESI and IECI groups maintained consistent sexual behaviour.

### 3.2 Semen Quality and Morphology

**FIGURE 7:**
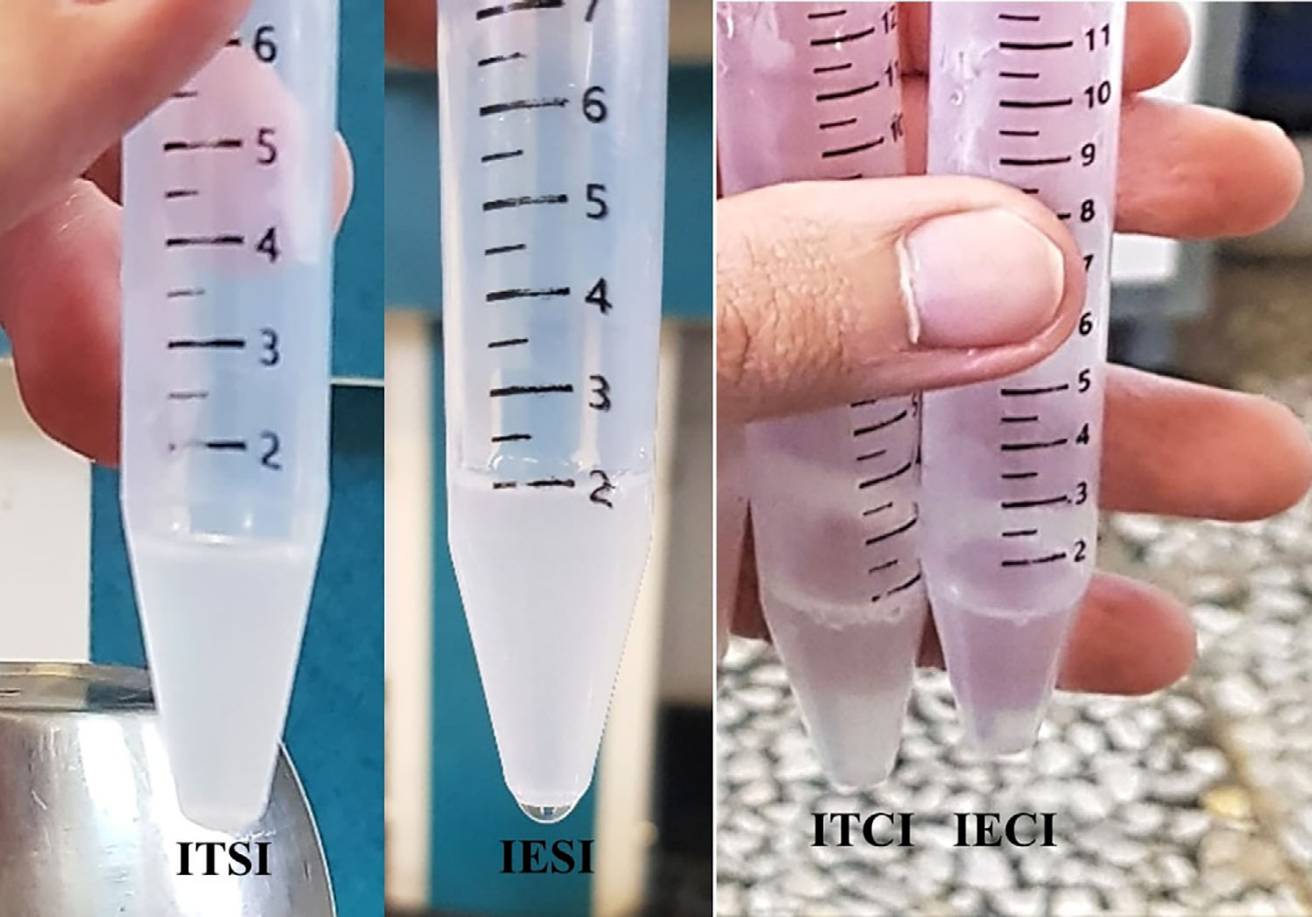
View of semen colour in each group (*n*:6) on D40. Semen colours in ITCI and IECI groups become clear on D40, whereas the other two control groups (ITSI and IESI) retained the natural milky colour.

**FIGURE 8:**
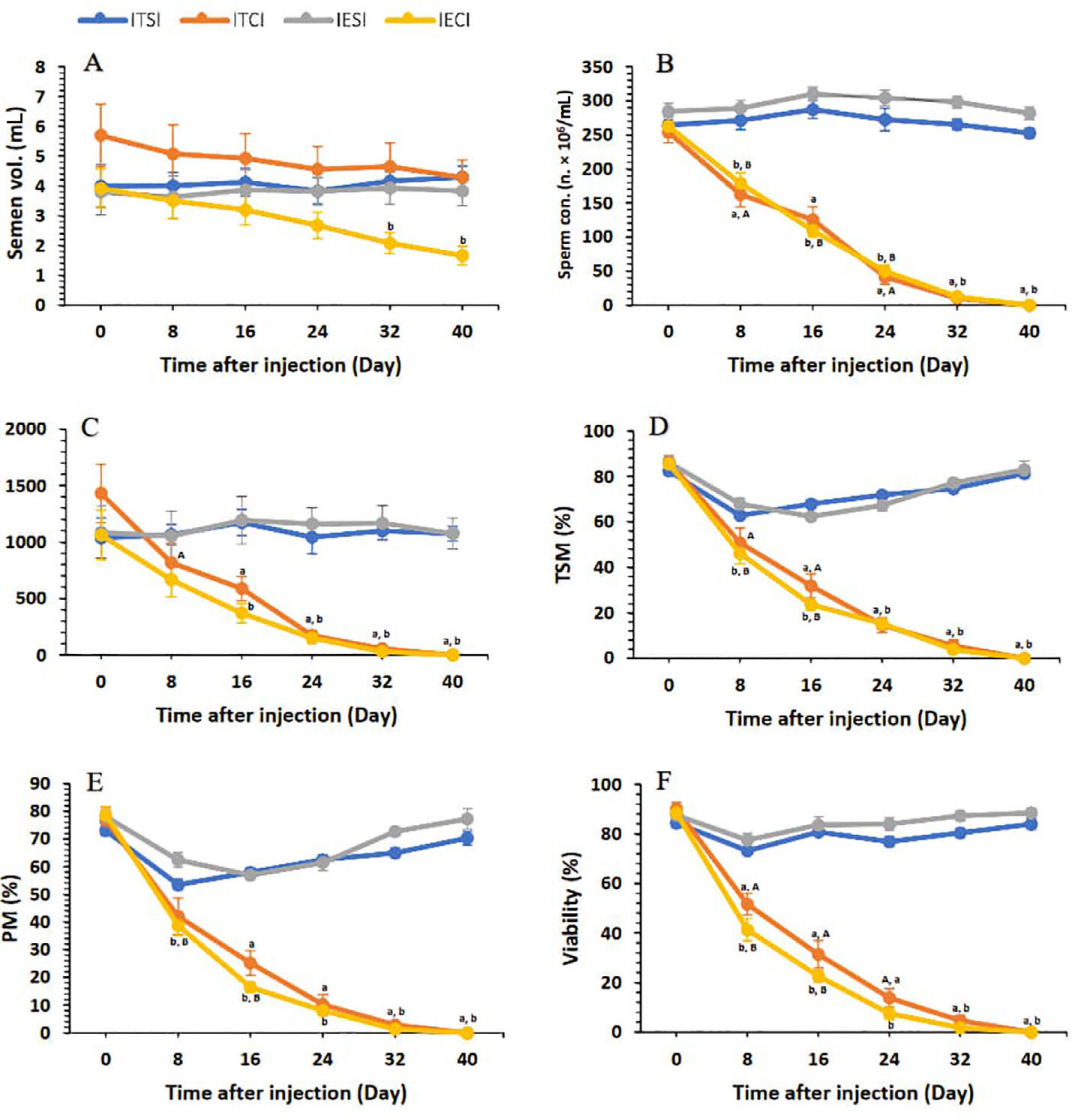
Change in semen volume (mL; A), concentration (*n* × 10^6^/mL; B), total sperm count (*n* × 10^6^; C), total sperm motility (%; D), progressive motility (%; E) and viability (%; F) in each group (*n* = 6) at different times (days) after intratesticular and intra‐epididymal injection. a and b values represent significant differences (*p* < 0.05) in intratesticular and intra‐epididymal injection, respectively, between control and treatment groups at the indicated times. A and B values represent significant (*p* < 0.05) time differences in intratesticular and intra‐epididymal injection, respectively, within treatment during the study. con, concentration; IECI, intra‐epididymal clove‐oil injection; IESI, intra‐epididymal saline injection; ITCI, intratesticular clove‐oil injection; ITSI, intratesticular saline injection; PM, progressive motility; TSC, total sperm count; TSM, total sperm motility; vol, volume.

**FIGURE 9:**
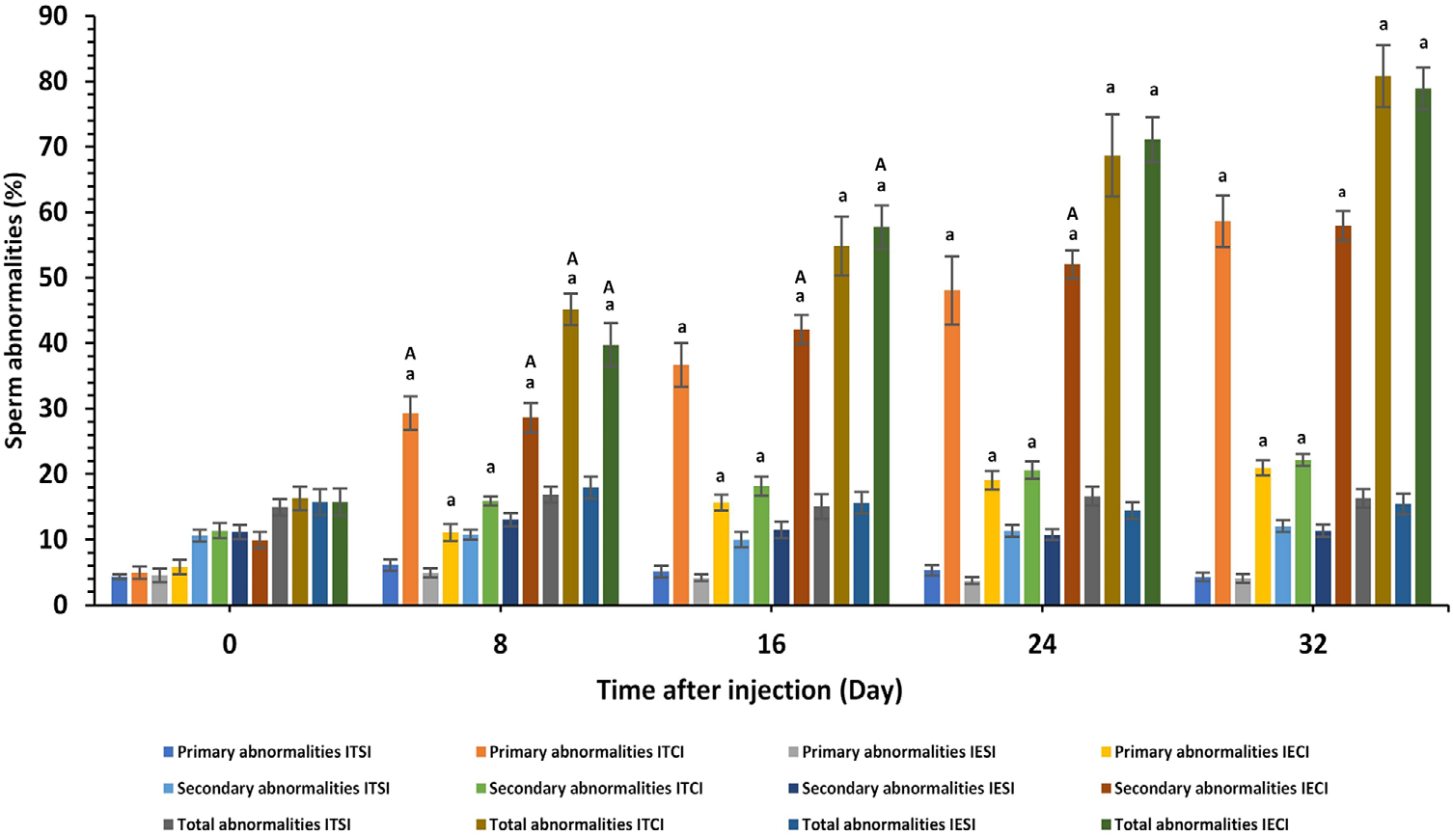
Change in primary, secondary and total abnormalities of sperm (%) in each group (*n* = 6) at different times (days) after intratesticular and intra‐epididymal injection. a value represents significant differences (*p* < 0.05) between control and treatment groups at the indicated times. A value represents significant (*p* < 0.05) time differences within treatment during the study. IECI, intra‐epididymal clove‐oil injection; IESI, intra‐epididymal saline injection; ITCI, intratesticular clove‐oil injection; ITSI, intratesticular saline injection.

Both ITI and IEI treatments led to an insignificant reduction in semen volume (mL) except for D32–D40 periods after IEI injection (*p* < 0.05) (Figure 8A). On Day 0, semen exhibited a milk‐like appearance; however, by Day 40, samples from the ITCI and IECI groups turned colourless, whereas those from the control groups remained milky (see Figure 7). Significant interactions between group and time were observed for total sperm count (×10^6^) and total sperm motility (%) during Days 16–40 (D16–D40) following ITI and IEI injection (*p* < 0.005; Figure 8C,D). From D8 to D40, sperm concentration (×10^6^/mL), viability (%) and progressive motility decreased (%), whereas primary, secondary and total abnormalities (%) increased in the ITCI and IECI groups, compared to the control groups (*p* < 0.005) (Figures 8B,E,F and 9).

### 3.3 Plasma Testosterone Levels

Serum testosterone levels, measured before and after HCG injection on Day 0, showed a significant increase from 3.08 ± 0.21 to 4.54 ± 0.39 ng/mL (*p* < 0.005; Table 2). Throughout the study, the ITCI group demonstrated a progressive decline in testosterone levels beginning on D3 and continuing through D40 (*p* < 0.0001), whereas the IECI group did not differ significantly from its control (IESI).

**TABLE 2:** Values are means ± SEM, with corresponding 95% confidence intervals calculated for each measurement.

		Serum testosterone (ng/mL)	
Time (D)/Group		Before HCG	After HCG
D0	ITSI	3.18 ± 0.26 (2.52–3.84)	4.85 ± 0.42 (3.76–5.94)^a^
	ITCI	3.40 ± 0.48 (2.17–4.63)	4.35 ± 1.17 (1.34–7.36)
	IESI	2.88 ± 0.40 (1.85–3.92)	4.38 ± 0.72 (2.53–6.24)
	IECI	2.85 ± 0.54 (1.47–4.23)	4.57 ± 0.83 (2.43–6.70)
D3	ITSI	—	4.75 ± 0.40 (3.71–5.79)
	ITCI	—	0.51 ± 0.10 (0.25–0.77)^b^, ^c^
	IESI	—	4.10 ± 0.63 (2.48–5.72)
	IECI	—	4.07 ± 0.63 (2.45–5.68)
D12	ITSI	—	4.70 ± 0.45 (3.55–5.85)
	ITCI	—	0.38 ± 0.09 (0.16–0.60)^b^
	IESI	—	4.12 ± 0.58 (2.62–5.61)
	IECI	—	3.85 ± 0.46 (2.66–5.04)
D21	ITSI	—	4.82 ± 0.44 (3.68–5.95)
	ITCI	—	0.45 ± 0.18 (0.00–0.92)^b^
	IESI	—	3.92 ± 0.75 (1.99–5.84)
	IECI	—	4.25 ± 0.58 (2.76–5.74)
D30	ITSI	—	4.87 ± 0.35 (3.95–5.78)
	ITCI	—	0.28 ± 0.07 (0.10–0.46)^b^
	IESI	—	3.93 ± 0.71 (2.11–5.75)
	IECI	—	4.40 ± 0.65 (2.72–6.08)
D40	ITSI	—	4.97 ± 0.35 (4.05–5.88)
	ITCI	—	0.11 ± 0.02 (0.49–0.17)^b^
	IESI	—	4.25 ± 0.71 (2.43–6.06)
	IECI	—	4.48 ± 0.77 (2.50–6.46)

*Note*: Generally, the treatment (treat group vs. con group) and time effect showed significant differences in serum testosterone levels (*p* < 0.0001) after intratesticular injection of clove oil.

Abbreviations: D, day; IECI, intra‐epididymal clove‐oil injection; IESI, intra‐epididymal saline injection; ITCI, intratesticular clove‐oil injection; ITSI, intratesticular saline injection; SEM, standard error of the mean.

^a^ Values represent significant differences (*p* < 0.05) between before and after HCG injection in each group on D0.

^b^ Value represents significant differences at *p* < 0.05 between control and treatment groups at the indicated times.

^c^ Value represents significant (*p* < 0.05) time differences within treatment during the study.

### 3.4 Ultrasound Findings on Testicular and Epididymal Changes

**FIGURE 10:**
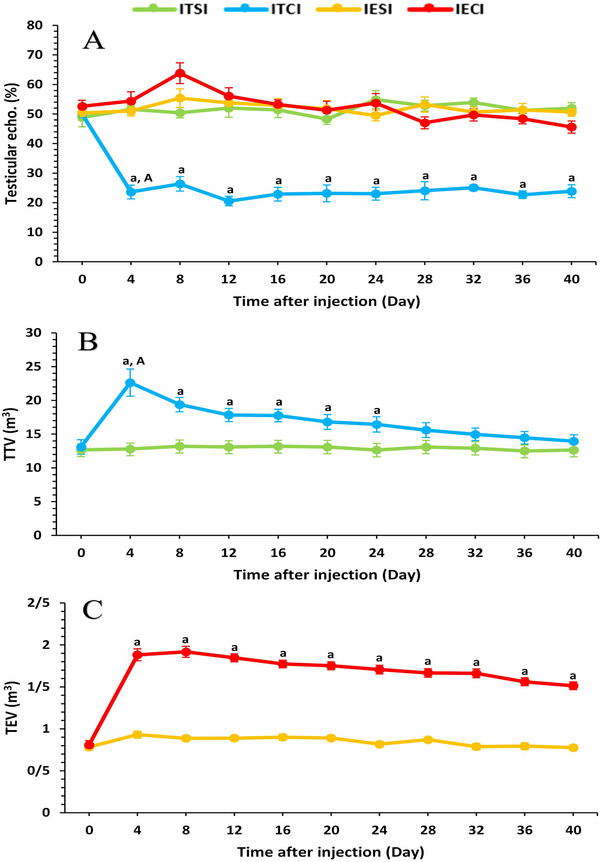
Effect of intratesticular and intra‐epididymal injection of clove oil on testicular echogenicity (%; A), total testicular volume (m^3^; B) and total epididymal tail volume (m^3^; C) measured in four groups (*n* = 6) on different days. a value represents significant differences (*p* < 0.05) between control and treatment groups at the indicated times. A value represents significant (*p* < 0.05) time differences within treatment during the study. echo, echogenicity; IECI, intra‐epididymal clove‐oil injection; IESI, intra‐epididymal saline injection; ITCI, intratesticular clove‐oil injection; ITSI, intratesticular saline injection; TEV, total epididymal tail volume; TTV, total testicular volume.

The ITCI group showed a significant increase in TTV (cm^3^) on D4 compared to ITSI (*p* < 0.005), followed by a gradual decrease from D8 to D24 (*p* < 0.05); differences were non‐significant during Days 28–40 (Figure 10B). In the IECI group, TEV (cm^3^) was significantly higher on Days 4 and 8 relative to IESI, with a gradual decline thereafter till D40 (*p* < 0.0001) (Figure 10C). The ITCI group experienced a significant decrease in testicular echogenicity (%) from Day 4 onwards compared to ITSI (*p* < 0.0001), whereas no significant differences were noted between the IECI and IESI groups (Figure 10A).

### 3.5 Doppler Blood Flow Parameters

The values of blood flow parameters, including PSV (cm/s), EDV (cm/s), RI and PI, for the four groups (ITSI, ITCI, IESI and IECI) during the study are presented in Table 3. Regarding the treatment × time interaction in ITI, significant differences were noted for all four parameters (*p* < 0.05). In contrast, for IEI, no significant differences were observed in blood flow parameters except for PSV (*p* < 0.0001). On D4, the PSV values of ITCI and IECI were significantly increased compared to ITSI and IESI. However, this value decreased during D28–D40 (*p* < 0.05; Table 3). In contrast, the PSV value for the IECI group decreased on D16 *(p* < 0.05). The EDV parameter decreased in the ITCI group compared to ITSI from D12 to D40 (*p* < 0.05), whereas in the IECI group, EDV was reduced compared to IESI on D8 (*p* < 0.05). The RI parameter increased in the ITCI group compared to ITSI from D16 to D40 (*p* < 0.05). In the IECI group, RI increased on D4 and D8 but decreased on D24 compared to IESI (*p* < 0.05). The PI parameter significantly increased from D16 to D40 in the ITCI group and on D4 and D32 in the IECI group compared to their control groups (*p* < 0.05).

**TABLE 3:** Values are means ± SEM, with corresponding 95% confidence intervals calculated for each measurement.

Time (D)/Group		PSV (cm/s)	EDV (cm/s)	RI	PI
D0	**ITSI**	23.50 ± 1.45 (19.76–27.24)	9.43 ± 1.54 (5.48–13.38)	0.61 ± 0.04 (0.51–0.71)	0.99 ± 0.14 (0.65–1.34)
	**ITCI**	22.93 ± 1.46 (19.18–26.69)	8.27 ± 1.19 (5.21–11.32)	0.65 ± 0.04 (0.54–0.75)	1.08 ± 0.15 (0.71–1.45)
	**IESI**	22.67 ± 0.85 (20.47–24.86)	9.07 ± 1.54 (5.11–13.04)	0.60 ± 0.07 (0.43–0.77)	1.15 ± 0.20 (0.63–1.66)
	**IECI**	21.87 ± 1.60 (17.75–25.98)	8.54 ± 1.30 (5.20–11.88)	0.61 ± 0.04 (0.50–0.72)	1.03 ± 0.14 (0.66–1.40)
D4	**ITSI**	26.62 ± 1.06 (23.90–29.34)	8.04 ± 0.43 (6.92–9.15)	0.70 ± 0.02 (0.66–0.74)	1.24 ± 0.06 (1.10–1.39)
	**ITCI**	36.93 ± 3.14 (28.87–45.00)^a^	9.88 ± 1.51 (6.00–13.77)	0.74 ± 0.02 (0.68–0.79)	1.36 ± 0.13 (1.02–1.71)
	**IESI**	28.07 ± 0.80 (26.00–30.13)	7.06 ± 0.47 (5.86–8.26)	0.75 ± 0.01 (0.71–0.79)	1.48 ± 0.14 (1.12–1.84)
	**IECI**	40.45 ± 1.50 (36.60–44.30)^[Link]^, ^[Link]^	7.47 ± 0.87 (5.22–9.71)	0.81 ± 0.02 (0.76–0.87)^[Link]^, ^[Link]^	2.17 ± 0.21 (1.62–2.72)^a^
D8	**ITSI**	22.70 ± 0.77 (20.73–24.67)	10.31 ± 1.34 (6.86–13.75)	0.55 ± 0.05 (0.41–0.68)	1.10 ± 0.15 (0.71–1.50)
	**ITCI**	24.83 ± 2.12 (19.39–30.27)	8.79 ± 1.74 (4.32–13.26)	0.66 ± 0.05 (0.52–0.80)	1.21 ± 0.15 (0.84–1.59)
	**IESI**	24.45 ± 0.83 (22.30–26.59)	7.17 ± 0.40 (6.14–8.20)	0.71 ± 0.02 (0.67–0.75)	1.08 ± 0.17 (0.65–1.51)
	**IECI**	25.40 ± 2.21 (19.72–31.08)	5.12 ± 0.75 (3.20–7.04)^a^	0.79 ± 0.03 (0.72–0.86)^a^	1.82 ± 0.33 (0.98–2.67)
D12	**ITSI**	23.98 ± 0.53 (22.62–25.34)	8.54 ± 0.49 (7.28–9.80)	0.64 ± 0.02 (0.59–0.70)	1.12 ± 0.19 (0.64–1.61)
	**ITCI**	21.02 ± 2.13 (15.54–26.50)	5.61 ± 0.85 (3.42–7.81)^a^	0.73 ± 0.05 (0.61–0.84)	1.40 ± 0.18 (0.94–1.97)
	**IESI**	23.52 ± 0.72 (21.64–25.36)	6.68 ± 0.39 (5.67–7.68)	0.72 ± 0.01 (0.69–0.75)	1.25 ± 0.17 (0.82–1.67)
	**IECI**	18.43 ± 2.33 (12.44–24.42)^b^	5.57 ± 0.60 (4.01–7.12)	0.67 ± 0.06 (0.51–0.83)	1.29 ± 0.21 (0.75–1.82)
D16	**ITSI**	22.68 ± 0.97 (20.18–25.19)	8.61 ± 0.39 (7.60–9.61)	0.62 ± 0.02 (0.57–0.67)	1.12 ± 0.17 (0.69–1.55)
	**ITCI**	23.77 ± 1.22 (20.63–26.90)	5.44 ± 0.65 (3.77–7.11)^a^	0.77 ± 0.02 (0.71–0.83)^a^	1.76 ± 0.12 (1.44–2.08)^a^
	**IESI**	22.25 ± 0.65 (20.59–23.91)	6.43 ± 0.33 (5.58–7.27)	0.71 ± 0.01 (0.68–0.75)	1.34 ± 0.15 (0.95–1.73)
	**IECI**	18.12 ± 1.67 (13.82–22.41)^a^	6.56 ± 1.21 (3.45–9.67)^a^	0.64 ± 0.04 (0.53–0.75)	1.29 ± 0.15 (0.90–1.69)
D20	**ITSI**	24.82 ± 1.11 (21.96–27.68)	9.47 ± 0.92 (7.11–11.84)	0.61 ± 0.05 (0.47–0.74)	0.94 ± 0.19 (0.46–1.42)
	**ITCI**	21.50 ± 1.76 (16.98–26.02)	4.56 ± 0.61 (2.98–6.13)^a^	0.78 ± 0.04 (0.69–0.87)^a^	1.90 ± 0.25 (1.26–2.55)^a^
	**IESI**	21.53 ± 0.57 (20.06–23.00)	7.28 ± 0.62 (5.69–7.21)	0.66 ± 0.03 (0.58–0.74)	1.22 ± 0.07 (1.04–1.39)
	**IECI**	20.33 ± 1.53 (16.40–24.26)	6.62 ± 0.86 (4.41–8.84)	0.67 ± 0.05 (0.53–0.80)	1.18 ± 0.14 (0.82–1.54)
D24	**ITSI**	22.48 ± 0.65 (20.82–24.86)	8.40 ± 0.29 (7.66–9.13)	0.63 ± 0.01 (0.59–0.67)	1.20 ± 0.11 (0.91–1.48)
	**ITCI**	20.50 ± 1.40 (16.91–24.09)	3.58 ± 0.84 (1.41–5.75)^a^	0.82 ± 0.04 (0.70–0.93)^a^	2.15 ± 0.27 (1.47–2.84)^a^
	**IESI**	22.68 ± 0.70 (20.90–24.47)	6.84 ± 0.31 (6.03–7.64)	0.70 ± 0.02 (0.66–0.74)	1.22 ± 0.11 (0.95–1.49)
	**IECI**	19.95 ± 2.00 (14.80–25.10)	6.87 ± 0.31 (6.06–7.68)	0.63 ± 0.05 (0.50–0.76)^a^	1.28 ± 0.18 (0.83–1.73)
D28	**ITSI**	23.15 ± 0.88 (20.89–25.41)	8.54 ± 0.65 (6.86–10.22)	0.63 ± 0.02 (0.57–0.69)	1.17 ± 0.16 (0.75–1.60)
	**ITCI**	18.02 ± 1.92 (13.08–22.95)^a^	3.25 ± 0.78 (1.25–5.25)^a^	0.82 ± 0.04 (0.70–0.93)^a^	2.31 ± 0.39 (1.31–3.31)^a^
	**IESI**	23.47 ± 0.60 (21.92–25.01)	6.76 ± 0.36 (5.82–7.70)	0.71 ± 0.01 (0.69–0.74)	1.26 ± 0.10 (1.01–1.52)
	**IECI**	21.57 ± 1.04 (18.88–24.25)	6.00 ± 1.13 (3.10–8.90)	0.72 ± 0.05 (0.60–0.85)	1.23 ± 0.24 (0.61–1.86)
D32	**ITSI**	22.25 ± 1.02 (19.62–24.88)	8.51 ± 0.54 (7.13–9.90)	0.61 ± 0.03 (0.53–0.70)	1.28 ± 0.12 (0.99–1.58)
	**ITCI**	15.65 ± 1.19 (12.60–18.70)^a^	2.43 ± 0.74 (0.52–4.34)^a^	0.84 ± 0.05 (0.70–0.97)^a^	2.21 ± 0.27 (1.52–2.91)^a^
	**IESI**	22.42 ± 0.49 (21.16–23.67)	6.94 ± 0.45 (5.79–8.08)	0.69 ± 0.02 (0.64–0.74)	0.89 ± 0.13 (0.56–1.22)
	**IECI**	22.50 ± 1.84 (17.76–27.24)	7.24 ± 1.32 (3.84–10.63)	0.65 ± 0.09 (0.40–0.89)	1.37 ± 0.36 (0.43–2.30)^a^
D36	**ITSI**	24.17 ± 1.19 (21.10–27.23)	8.96 ± 1.65 (4.73–13.19)	0.63 ± 0.07 (0.45–0.80)	1.36 ± 0.28 (0.64–1.08)
	**ITCI**	12.80 ± 1.38 (9.26–16.34)^a^	1.46 ± 0.33 (0.62–2.30)^a^	0.87 ± 0.04 (0.78–0.96)^a^	2.72 ± 0.27 (2.02–3.43)^a^
	**IESI**	23.55 ± 0.71 (21.74–25.36)	8.10 ± 1.46 (4.36–11.84)	0.66 ± 0.06 (0.52–0.80)	1.19 ± 0.21 (0.64–1.74)
	**IECI**	23.08 ± 2.10 (17.69–28.47)	7.63 ± 1.15 (4.68–10.58)	0.69 ± 0.05 (0.57–0.80)	1.28 ± 0.26 (0.63–1.94)
D40	**ITSI**	23.02 ± 1.43 (19.33–26.70)	8.81 ± 1.13 (5.91–11.72)	0.62 ± 0.03 (0.55–0.69)	1.27 ± 0.14 (0.90–1.64)
	**ITCI**	10.99 ± 0.93 (8.59–13.38)^a^	0.98 ± 0.28 (0.27–1.68)^a^	0.91 ± 0.03 (0.84–0.98)^a^	3.05 ± 0.32 (2.22–3.88)^a^
	**IESI**	23.28 ± 1.22 (20.15–26.41)	9.08 ± 1.70 (4.70–13.46)	0.62 ± 0.05 (0.49–0.76)	1.17 ± 0.24 (0.56–1.77)
	**IECI**	23.20 ± 1.25 (19.99–26.41)	9.00 ± 1.40 (5.39–12.61)	0.61 ± 0.06 (0.48–0.76)	1.19 ± 0.19 (0.70–1.67)

*Note*: The treatment effect (treat group vs. con group) showed significant differences in PSV (cm/s), EDV (cm/s), RI and PI (*p* < 0.05) after intratesticular and intra‐epididymal injection of clove oil. The time effect showed significant differences in PSV, RI and PI in intra‐epididymal injections and in PSV, EDV, RI and PI in intratesticular injections (*p* < 0.01).

Abbreviations: D, day; EDV, end diastolic velocity (cm/s); IECI, intra‐epididymal clove‐oil injection; IESI, intra‐epididymal saline injection; ITCI, intratesticular clove‐oil injection; ITSI, intratesticular saline injection; PI, pulsatility index; PSV, peak systolic velocity; RI, resistive index; SEM, standard error of the mean.

^a^Value indicates significant differences (*p* < 0.05) between control and treatment groups at the indicated times.

^b^Value indicates significant (*p* < 0.05) time differences within treatment during the study.

### 3.6 Morphometric Indices

**FIGURE 11:**
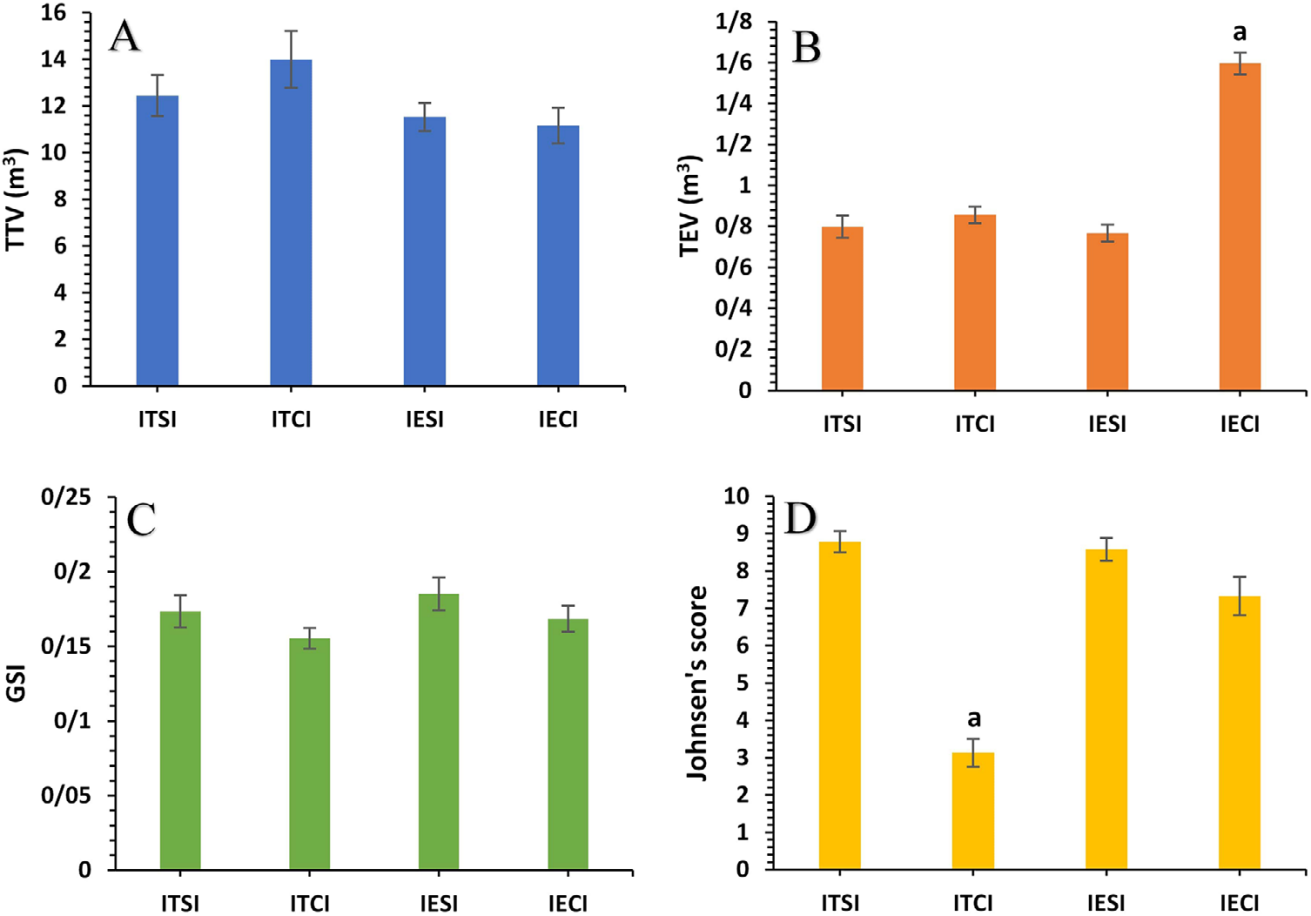
Change in total testicular volume (m^3^; A) and total epididymal tail volume (m^3^; B) measured by caliper, gonadosomatic index (C) and Johnsen's score (D) in each group (*n* = 6) on Day 40 after intratesticular and intra‐epididymal injection. a value represents significant differences (*p* < 0.05) between control and treatment groups on Day 40. GSI, gonadosomatic index; IECI, intra‐epididymal clove‐oil injection; IESI, intra‐epididymal saline injection; ITCI, intratesticular clove‐oil injection; ITSI, intratesticular saline injection; TEV, total epididymal tail volume; TTV, total testicular volume.

The mean TTV (cm^3^) in the ITCI and IECI groups did not change significantly compared to their respective control groups (Figure 11A). However, the mean TEV (cm^3^) in the IECI group was significantly higher than in the IESI group (*p* < 0.005). In contrast, no significant changes were observed in the ITCI group compared to the ITSI group (Figure 11B). Similarly, the GSI showed no significant decrease in the treatment groups compared to their control groups, consistent with the findings for TTV (Figure 11C).

### 3.7 Histopathology and Tissue Morphology

**FIGURE 12:**
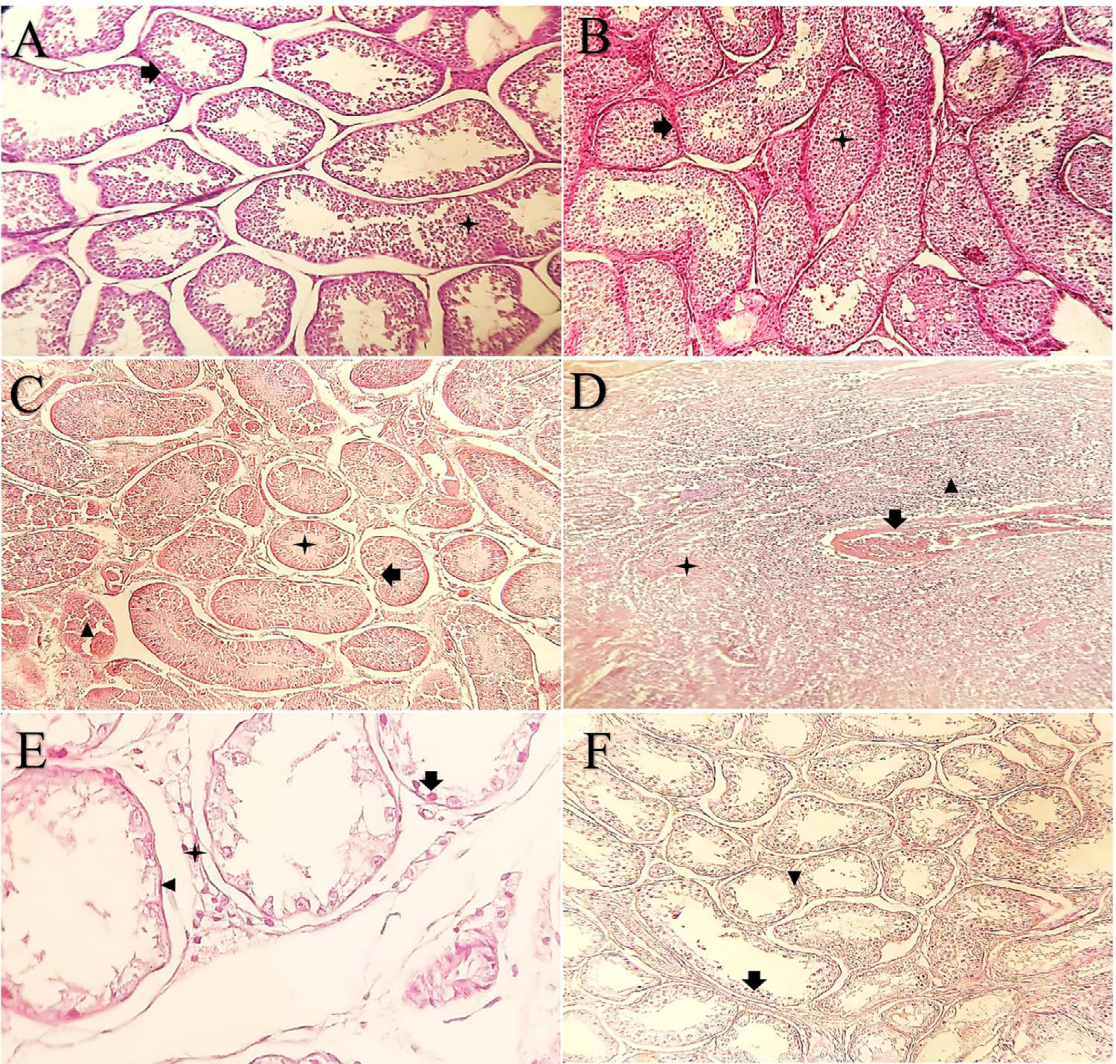
Histological changes of testis in each group after intratesticular and intra‐epididymal injection at D40. (A) ITSI group and (B) IESI group, arrangement of germ cells in the seminiferous tubules, indicating normal spermatogenesis (star) with no notable damage, and distinct interstitial space (arrow) was observed in each group (H&E, 200×). (C) ITCI group, atrophied seminiferous tubules showing luminal accumulation of necrotic debris and loss of normal histological architecture (star), accompanied by germ cell degeneration (arrow head). A few tubules retained lumens partially filled with germ cells and spermatozoa (arrow) (H&E, 100×). (D) ITCI group, complete necrosis of testicular parenchyma without any distinctive boundary between tubules (star) as well as the presence of fibrous tissue (arrow) and inflammatory cells (arrow head) was observed (H&E, 100×). (E) ITCI group, marked reduction in germ cell number (arrow) and thinning of the germinal epithelium (arrow head), with poorly defined boundaries between seminiferous tubules (star) (H&E, 400×). (F) IECI group, there was a decrease in number of germ cells (arrow) and thickness of germinal epithelium (arrow head), although these changes were not significant.

**FIGURE 13:**
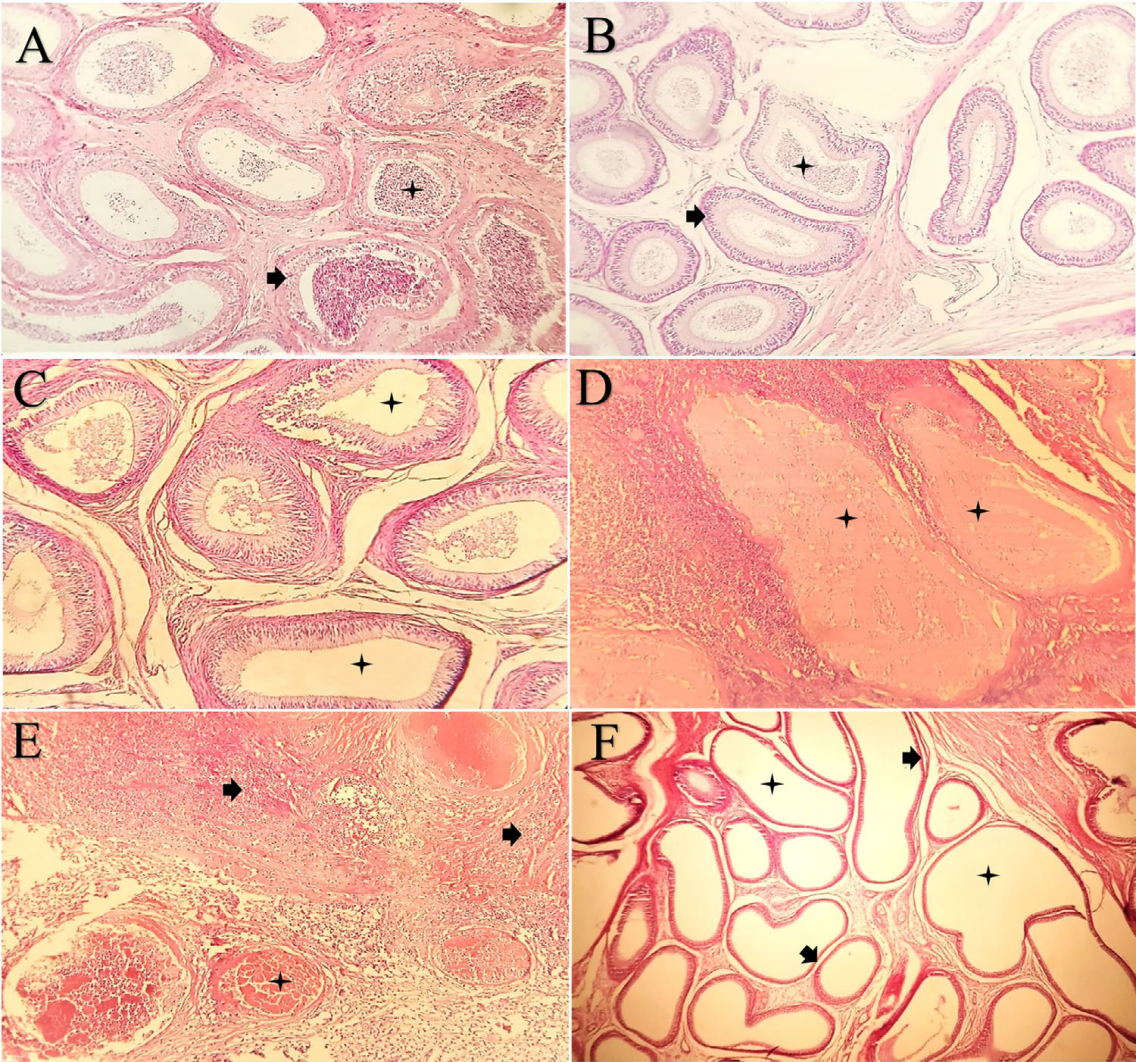
Histological changes of cauda (tail) epididymis in each group after intratesticular and intra‐epididymal injection at D40. (A) ITSI group and (B) IESI group, no change in epithelium of coils (arrow) and presence of normal sperm (star) was observed (H&E, 100×). (C) ITCI group, columnar epithelium coils that some lumen had no sperm were noticed (star) (H&E, 200×). (D) IECI group, complete histological degeneration of tubules’ appearance and caseous necrosis in lumen (star) was observed (H&E, 200×). (E) IECI group, accumulation of inflammatory cells could be recognized (arrow) as well as most of the area was lined by fibrous connective tissue (star) (H&E, 200×). (F) IECI group, histology of mid‐section of epididymis. Flattening with a marked reduction in the height of the columnar epithelium (arrow), along with the absence of sperm within the coils (star), was noticed (H&E, 40×).

The diameter of seminiferous tubules, the distance between tubules, the thickness of the germinal layer and the numbers of Leydig cells, Sertoli cells, spermatogonia, spermatocytes and spermatids in the treatment and control groups are summarized in Table 4. The diameter of seminiferous tubules in the ITCI group was significantly smaller than in the ITSI group. Conversely, the diameter in the IECI group was larger than in the IESI group, though this difference was not statistically significant. The thickness of the germinal layer was significantly lower in the ITCI group compared to the ITSI group. A similar trend was observed in the IECI group compared to the IESI group, but this difference was not significant. The distance between tubules was wider in the ITCI and IECI groups than in their respective controls; the increase was significant in the ITCI group. In the ITCI group, the number of Leydig, Sertoli, spermatogonia, spermatocytes and spermatids were significantly lower than that of the ITSI group. Although these values were also lower in the IECI group than in the IESI group, the differences were not statistically significant. Johnsen's score was significantly lower in the ITCI group (3.13 ± 0.37) compared to the ITSI group (8.78 ± 0.28; *p* < 0.0001), whereas no significant difference was observed between the IECI and IESI groups (Figure 11D). Microscopic examinations of samples from the ITSI and IESI groups preserved the normal structure of the testis and capsule, showing no changes in the cellular structure of the testis (Figure 12A,B). Evidence of sperm presence in the lumen of seminiferous tubules and the multilayered germinal epithelium was observed at 400× magnification (Figure 12B). Following ITI injection of pure clove essential oil, pervasive necrosis was observed throughout the testicular tissue, including tissue infarction (Figure 12C). The seminiferous tubular architecture exhibited complete disruption, with both tubular and interstitial components replaced by fibrous tissue, as seen at 400× magnification. The walls of these ducts were lined with a long columnar epithelium (Figure 13C). As shown in Figure 12E, there was a decrease in the number of germ cells, accompanied by a thinning of the germinal epithelium layer. Furthermore, complete necrosis of the testicular parenchyma was observed, accompanied by marked inflammatory cell infiltration and scar formation characterized by fibroblast proliferation and deposition of newly formed collagen fibres (Figure 12D). No significant changes were observed in the structure of epididymal ducts or the number of sperm in the lumen for the ITSI and IESI groups (Figure 13A,B). Following the IEI injection of pure clove extract, there was a non‐significant decrease in the number of germ cells and the thickness of the germinal epithelium. Many seminiferous tubule lumens lacked sperm (Figure 12F). Histological sections from the IECI group demonstrated an absence of sperm in the coils, decreased height of the columnar epithelium and dilation of the epididymal ducts (Figure 13F). Degeneration of the tubules and caseous necrosis were observed in many slides, rendering the tubules undetectable (Figure 13D). The accumulation of inflammatory cells and the formation of fibrous tissue were also evident (Figure 13E). According to Table 4, the ITCI group showed significant reductions in the diameter of seminiferous tubules (µm), the thickness of the germinal epithelium (µm) and the numbers of Sertoli cells, Leydig cells, spermatogonia, primary spermatocytes and spermatids compared to the ITSI group after ITI injection of clove oil on D40 (*p* < 0.05). The distance between seminiferous tubules increased significantly (µm) in the ITCI group compared to the ITSI group (*p* < 0.05). On the other hand, there were no significant differences in the diameter of seminiferous tubules, the thickness of the germinal epithelium, the number of Sertoli cells, Leydig cells, spermatogonia, primary spermatocytes or spermatids between the IECI and IESI groups following IEI clove‐oil injection on D40 (*p* > 0.05; Table 4).

**TABLE 4:** Changes in seminiferous tubule diameter (µm), germinal epithelium thickness (µm), seminiferous tubule diameter (µm) and the number of Sertoli cells, Leydig cells, spermatogonia, primary spermatocytes and spermatids in each group (*n* = 6) on Day 40 after intratesticular and intra‐epididymal injection.

Group	ITSI	ITCI	IESI	IECI
Diameter of seminiferous tubule (µm)	189.50 ± 5.30 (175.88–203.12)	171.42 ± 3.13 (163.37–179‐47)^a^	186.6 ± 5.02 (173.71–199.51)	187.38 ± 4.23 (176.50–198.25)
Germinal epithelium thickness (µm)	50.52 ± 3.50 (41.52–59.51)	18.42 ± 1.16 (15.42–21.41)^a^	49.83 ± 3.53 (40.45–58.90)	40.96 ± 2.60 (34.28–47.64)
Distance between seminiferous tubules (µm)	19.52 ± 1.49 (15.67–23.36)	56.25 ± 2.68 (49.37–63.13)^a^	14.25 ± 1.24 (11.05–17.45)	16.00 ± 1.98 (10.90–21.10)
Sertoli cell	10.13 ± 0.42 (9.06–11.21)	3.45 ± 0.46 (2.26–4.64)^a^	8.25 ± 0.56 (6.81–9.69)	6.41 ± 0.68 (4.67–8.15)
Leydig cell	5.07 ± 0.21 (4.52‐5.61)	2.11 ± 0.26 (1.45–2.77)^a^	5.31 ± 0.27 (4.61–6.01)	5.24 ± 0.47 (4.02–6.46)
Spermatogonia	30.85 ± 1.24 (27.67–34.03)	5.03 ± 0.70 (3.24–6.83)^a^	28.60 ± 1.37 (25.07–32.13)	22.73 ± 2.61 (16.03–29.43)
Spermatocyte	34.20 ± 1.13 (31.30–37.10)	3.04 ± 0.63 (1.41–4.67)^a^	32.35 ± 1.28 (29.07–35.63)	26.02 ± 2.57 (19.41–32.63)
Spermatids	28.48 ± 1.00 (25.91–31.06)	2.06 ± 0.28 (1.35–2.77)^a^	27.03 ± 1.77 (22.48–31.59)	21.25 ± 2.35 (15.21–27.29)

*Note*: Values are means ± SEM, with corresponding 95% confidence intervals.

Abbreviations: IECI, intra‐epididymal clove‐oil injection; IESI, intra‐epididymal saline injection; ITCI, intratesticular clove‐oil injection; ITSI, intratesticular saline injection; SEM, standard error of the mean.

^a^ Value represents significant differences (*p* < 0.05) between control and treatment groups on Day 40.

## 4 Discussion

For decades, veterinarians have relied on surgical techniques to control dog populations by limiting breeding (Moldave and Rhodes 2013). Although effective, these procedures are associated with high financial costs, prolonged recovery, animal stress and increased post‐operative risks (Adin 2011; Neilson et al. 1997). Chemical sterilization emerged as a promising alternative due to its ability to circumvent these limitations. This approach aims to leverage technological innovations to establish a sterilization method that is more cost‐effective, technically straightforward, characterized by low‐invasiveness and associated with fewer complications than conventional surgical castration, thereby mitigating the inherent limitations of standard surgical procedures. The present study evaluated the efficacy of pure clove essential oil as a chemical sterilant, comparing ITI and IEI administration routes. Unlike many previously tested agents (Seid and Terefe 2019), clove oil contains eugenol, which induces peroxidative damage and tissue degeneration via free radical activity (Abshenas et al. 2013; Kumar Jaganathan and Supriyanto 2012). Our objective was to determine whether clove oil could reduce spermatogenesis and impair semen quality without causing excessive tissue damage. Early chemical sterilants, such as calcium chloride and zinc gluconate, have demonstrated variable outcomes, with low doses failing to achieve permanent sterilization and higher doses causing adverse reactions, including inflammation, ulcers and fistula formation (Levy et al. 2008; Seid and Terefe 2019). To provide a clearer comparative framework, Table 5 synthesizes the mechanisms, reported effects, merits and associated limitations of clove oil, calcium chloride and zinc gluconate. Our findings suggest that clove oil achieves comparable sterilization while producing moderate and self‐limiting local tissue reactions, making it a potentially safer alternative.

**TABLE 5:** Comparative characteristics of clove essential oil, calcium chloride and zinc gluconate used as chemical sterilants in male dogs.

Sterilant	Technique (route)	Mechanism of action	Reported outcomes	Tissue effects	Advantages	Disadvantages
Clove essential oil	Intratesticular	Eugenol triggers free‑radical‑mediated cytotoxicity → seminiferous tubular necrosis → permanent infertility	Marked ↓testosterone, azoospermia	Severe necrosis, fibrosis	Irreversible testicular necrosis; simple administration; strong sterilizing effect	Transient swelling
	Intra‑epididymal	Chemical irritation → epithelial degeneration → sperm transport obstruction	↓Motility, ↓viability	Duct dilation, mild degeneration	Minimal effects on testosterone; avoids full testicular destruction	Procedurally demanding; requires ultrasound guidance
Calcium chloride	Intratesticular	Causes sclerosis of seminiferous epithelium → progressive testicular atrophy	Azoospermia, ↓testosterone	Degeneration, fibrosis	Low‑cost; practical for field conditions	Can cause pain, inflammation and ulceration if solution concentration is high
	Intra‑epididymal	Irritates and collapses epididymal tubules → sperm maturation/storage failure	Reduced sperm transit, ↓motility	Ductal fibrosis, luminal obstruction	Allows targeted obstruction of epididymal tail	Higher risk of local inflammation and inconsistent azoospermia
Zinc gluconate	Intratesticular	Zinc causes cellular toxicity and fibrosis of germinal tissue → permanent sterility	Permanent infertility in many dogs	Degeneration, interstitial fibrosis	Used in large‑scale sterilization campaigns; relatively safe at proper dilution	Risk of scrotal irritation, abscess or fistula in improper application
	Intra‑epididymal	Chemical sclerosis of epididymal tubules → sperm maturation arrest	Impaired maturation, ↓concentration	Macrophage infiltration, ductal damage	Selective effect on sperm storage region	Technique less standardized; data limited in dogs

*Note*: The table summarizes administration routes, mechanisms of action, reported effects on fertility and testicular tissue, advantages and disadvantages for each compound (Abshenas et al. 2013; Fahim et al. 1993; Leoci et al. 2019; Mohamed et al. 2024; Oliveira et al. 2012).

Semen quality was significantly impaired following clove‐oil administration. In both ITCI and IECI groups, the colour of semen shifted from white to clear by Day 40, consistent with oligospermia/azoospermia. Quantitative semen analysis confirmed a marked decline in sperm count and motility, with a significant increase in morphological abnormalities (*p* < 0.001). These results align with previous studies reporting similar outcomes after chemical sterilization (Maadi et al. 2021; Mohamed et al. 2024; Oliveira et al. 2012). ITI injections of chlorhexidine gluconate (Virendra et al. 2022) and formalin (Moustafa et al. 2023) successfully induced azoospermia and castration. In a similar manner, IEI administration of calcium chloride and zinc gluconate in dogs (Fahim et al. 1993; Leoci et al. 2019), as well as copper in mice (Xu et al. 1985), has been shown to significantly impair sperm concentration, viability, motility and chromatin integrity, primarily as a result of epididymal tissue damage (Talebi et al. 2007). Testicular degeneration impairs sperm morphology and functionality by elevating the incidence of abnormalities (Boudou et al. 2014; Oguejiofor et al. 2020). Primary defects originate during spermatogenesis, whereas secondary defects develop during epididymal maturation (Câmara et al. 2014). Experimental findings indicate that ITI injections predominantly induce primary abnormalities, while IEI injections primarily result in secondary alterations. In dogs, calcium chloride injections significantly increased abnormal sperm morphology by Day 28 compared with Day 0 (Mohamed et al. 2024). IEI zinc gluconate injections resulted in a higher prevalence of secondary abnormalities compared to primary ones (Fahim et al. 1993). IEI injection significantly reduced semen volume in the treatment group compared to the control group between Days 32 and 40, though decreased sperm concentration did not significantly alter global semen volume due to prostatic secretions (M. A. Kutzler 2005). ITI calcium chloride injection in bulls (Canpolat et al. 2006) and IEI zinc gluconate injection in dogs (Fahim et al. 1993) had no effect on semen volume. In dogs, semen volume is modulated by hormonal changes, reproductive activity, nutrition, stress, temperature and season (Tesi et al. 2018; Vágenknechtová et al. 2011).

ITI injections of clove‐oil extract, calcium chloride and zinc gluconate significantly reduced serum testosterone and sexual activity in dogs, as supported by multiple studies (Abshenas et al. 2013; AbuAhmed 2015; Jana and Samanta 2007; Rafatmah et al. 2019). No alterations in serum testosterone levels were observed in dogs treated with IEI clove extract, likely due to the absence of damage to parenchymal Leydig cells responsible for testosterone secretion. Similarly, IEI zinc gluconate and calcium chloride injections did not result in significant changes in testosterone concentration. (Fahim et al. 1993; Leoci et al. 2019). Sexual behaviours in the IEI clove injection (IECI) group remained unchanged through day 40, consistent with baseline. These findings underscore a strong correlation between serum testosterone levels and sexual behaviour in dogs (Leoci et al. 2014).

ITI administration of clove oil resulted in a transient increase in testicular volume due to mild swelling, followed by a significant reduction by Day 40. Zinc gluconate injected intratesticularly caused a slight increase in the size of tunica albuginea 2 weeks after treatment. This finding is consistent with the acute phase (15 days post‐treatment) and the early inflammatory response observed in the aforementioned histopathological studies (Oliveira et al. 2012). Compared with calcium chloride, clove oil induced a moderate and transient inflammatory response while avoiding pain or irritation, indicating a more favourable safety profile as reported by AbuAhmed (2015). Histopathological evidence demonstrated degenerative alterations associated with decreased testicular size and compromised semen quality (Kumar et al. 2023). In pre‐pubertal dogs, IEI zinc gluconate injections induced macrophage and leukocyte infiltration with histopathological changes evident by Day 10 (Chaudhary et al. 2018). Comparable inflammatory reactions in the epididymis following IEI injections of clove essential oil, characterized by leukocyte infiltration and luminal obstruction, may further contribute to sterility by obstructing sperm transport and highlight inflammation as a key mechanism in infertility (Leoci et al. 2019; Park et al. 2014).

By Day 40, testicular and epididymal tail volumes remained unchanged in the ITCI group compared with the ITSI group, whereas IEI injection increased epididymal volume, indicating localized effects. Kulkarni et al. (2024) showed that bilateral ITI zinc gluconate with DMSO reduced testicular dimensions and scrotal size and caused parenchymal degeneration, whereas the addition of lignocaine hydrochloride 1% prevented these changes (Kulkarni et al. 2024). Neither injection affected the GSI, suggesting that clove oil's impact on gonadal mass may require longer exposure, consistent with previous reports of delayed GSI reduction following ITI injection of saline and mannitol (Maadi et al. 2021).

Ultrasonographic and histological evaluations of the ITCI group showed reductions in testicular volume, increased echogenicity and degenerative alterations in seminiferous tubules, germinal layers and germ cells. A concomitant decline in Johnsen's score further emphasized impaired spermatogenesis. Collectively, these findings confirm that ITI clove‐oil injections induce significant morphological and functional damage to testicular tissue. Abshenas et al. (2013) reported that intra‐testicular injection of diluted clove oil in DMSO impaired germ cell proliferation, damaged seminiferous tubules and induced scarring (Abshenas et al. 2013). Sonography confirmed tissue injury, as evidenced by increased echogenicity (Leoci et al. 2019), reduced testicular volume and impaired spermatogenic function (Mohamed et al. 2024; Rafatmah et al. 2019). By Day 45, clove oil combined with lignocaine produced the most pronounced reduction in testicular volume, followed by eugenol, clove oil alone and clove oil with olive oil, whereas control animals exhibited minimal change. These results corroborate the association between these treatments and testicular injury, reflecting underlying structural alterations (Kumar et al. 2023). Epididymal administration of clove‐oil extract produced no discernible testicular histopathological or ultrasonographic changes, indicating preservation of testicular architecture; however, it caused noticeable alterations in the epididymal tissue. Taken together, these findings indicate that IECI induced functional alterations with limited structural disruption at day 40, although long‐term reversibility remains to be established. IEI zinc gluconate injections in pre‐pubertal dogs elicited marked fibrous proliferation, macrophage infiltration, hyaline degeneration of the cauda epididymis and tubular degeneration, while sparing the seminiferous tubules, suggesting a predominantly epididymal site of action (Chaudhary et al. 2018).

Analysis of testicular artery blood flow parameters serves as a diagnostic tool for assessing fertility and infertility in animals. Subcutaneous melatonin administration in male goats results in increased PSV and EDV, alongside decreased RI and PI, indicating enhanced testicular arterial blood flow and improved fertility (Samir et al. 2020). Similarly, higher PSV and EDV values are observed in fertile compared to infertile dogs, emphasizing these parameters’ relevance in fertility evaluation (Zelli et al. 2013). Mohamed et al. (2024) demonstrated that ITI injection of calcium chloride in dogs significantly increases RI and PI while decreasing PSV and EDV over 4 weeks, reflecting the impact of chemical sterilization on testicular blood flow and associated fertility indices (Mohamed et al. 2024). Similarly, contrast‐enhanced ultrasound has been used to document marked reductions in testicular perfusion immediately after ITI calcium chloride injection in dogs, further supporting the close relationship between chemical castration, vascular impairment and testicular dysfunction (Cicirelli et al. 2022). Doppler ultrasound parameters of the testicular artery in dogs correlate with histological evidence of testicular dysfunction, including parenchymal ATP damage, reduced tubular area and decreased seminiferous epithelium thickness, associated with slight increases in PI and RI (Gloria et al. 2020). ITI injection of clove oil led to increased RI and PI and decreased PSV and EDV values up to Day 40, indicating reduced testicular blood flow and impaired fertility. In contrast, IEI injection of clove oil at Days 20 and 40 does not significantly alter RI, PI or EDV, suggesting minimal effects on testicular vascularity, spermatogenesis and fertility. Additionally, regression analysis reveals that dog weight significantly influences PI and RI, with testicular depth positively correlated with weight, while testicular height shows no such correlation (Stefanizzi et al. 2022). These findings highlight that velocimetric indices reflect multiple physiological and anatomical factors affecting testicular blood flow and function.

This study has several limitations that should be acknowledged. A formal a priori power analysis was not conducted, and the relatively small sample size (24 dogs; four groups of six) may have reduced the precision and statistical power of some comparisons. The sample size was determined with reference to comparable published studies and ethical guidelines aimed at minimizing the use of animals (Fahim et al. 1993; Hamedi et al. 2018; Mohamed et al. 2024; Oliveira et al. 2012). Where possible, we now report 95% confidence intervals for key outcomes to provide a clearer indication of estimate precision. In addition, the study was conducted under unfavourable circumstances, which imposed time and financial constraints and limited the possibility of recruiting additional animals. Another limitation is the use of mixed‐breed dogs, which may have introduced physiological and histological variability, potentially affecting the consistency and generalizability of the results. Although random allocation was employed to minimize bias, inter‐individual heterogeneity remains an important consideration.

Another important limitation is the short follow‐up period (40 days), which constrains interpretation of long‐term fertility outcomes, regenerative capacity—particularly in the IECI group—and the potential for delayed adverse tissue effects. Longer follow‐up (e.g., 3–6 months) is necessary to determine whether the observed functional and structural changes, especially after IEI treatment, are fully reversible, partially reversible or permanent.

Future research should address these limitations by enrolling larger and more homogeneous populations, supported by formal sample size calculations, to enhance the robustness and external validity of findings. Longitudinal studies are warranted to corroborate these results and to better characterize the temporal progression of testicular and epididymal changes. Further methodological refinements, including optimization of dosages, refinement of injection techniques and exploration of combination therapies, may improve the safety and efficacy of clove‐oil‐based chemical sterilization. Additionally, the development of advanced approaches for assessing sperm characteristics would strengthen the evaluation of fertility outcomes. Finally, studies conducted under field conditions, ideally without general anaesthesia and long‐term follow‐up, are crucial to assess the practicality, welfare implications and potential reversibility of adverse effects associated with this sterilization method.

## 5 Conclusion

ITI and IEI administration of clove essential oil may represent cost‐effective and low‐invasiveness methods for male dog sterilization. These methods may reduce surgical burden and perioperative stress compared with orchiectomy, which could be advantageous in settings where access to surgery is limited. However, the IEI procedure is time‐consuming and generally requires ultrasonographic guidance, potentially limiting its field applicability. At Day 40, ITI administration produced extensive necrosis and fibrous replacement of testicular parenchyma, supporting its use as a permanent sterilization method, whereas IEI administration primarily affected epididymal structure and function, with testicular architecture largely preserved but with long‐term reversibility still requiring further investigation. These characteristics are particularly relevant for addressing dog overpopulation in both stray and owned populations while considering animal welfare and resource constraints. Collectively, our findings support clove oil as a promising and practical alternative to surgical castration in the short term and highlight the need for longer‐term follow‐up studies to define durability and safety.

## Author Contributions

**Morteza Poormohammad**: conceptualization, software, validation, formal analysis, investigation, resources, data curation, funding acquisition, project administration, writing – original draft preparation. **Mohammad Hossein Safari**: validation, resources, visualization, funding acquisition. **Shadi Emami Moghadam**: resources, validation, funding acquisition. **Ourang Ataie Amarloie**: methodology, supervision, visualization, investigation, resources, writing – review and editing. **Mehran Farhoodi Moghadam**: formal analysis, methodology, resources, data curation, validation. **Pegah Valitabar**: visualization, writing – review and editing. **Fariborz Moayer**: visualization, resources, investigation.

## Funding

The authors have nothing to report.

## Ethics Statement

The present study was executed in full compliance with the ethical guidelines established by the Institutional Animal Care and Use Committee. Before initiation, informed consent was obtained from all participating animal shelters in Alborz Province. The research protocol was reviewed and approved by the Islamic Azad University–Karaj Branch Ethics Committee (Approval ID: IR.IAU.K.REC.1399.053), and all procedures were conducted in accordance with the Principles for the Care and Use of Research Animals described in Directive 2010/63/EU for European Community animal experiments. Adequate anaesthesia and analgesia were provided before, during and after intratesticular and intra‐epididymal injections to minimize discomfort, and animals were monitored post‐procedurally for potential adverse effects. Although intratesticular injection of clove oil induces irreversible testicular necrosis, the method was chosen as a potential alternative to surgical castration, aiming to reduce surgical trauma, costs and recovery time. The ethical justification lies in its potential to provide a practical and less invasive tool for population control, particularly in free‐roaming and shelter dogs, where surgical sterilization is often not feasible.

## Conflicts of Interest

The authors declare no conflicts of interest.

## Data Availability

The data that support the findings of this study are available from the corresponding author upon reasonable request.
